# Combined multiple testing of multivariate survival times by censored empirical likelihood

**DOI:** 10.1111/sjos.12423

**Published:** 2019-12-17

**Authors:** Judith H. Parkinson

**Affiliations:** ^1^ Department of Mathematics University of Salzburg

**Keywords:** conditional hazards, multiple constraints, multivariate survival analysis, weighted log‐rank test

## Abstract

In each study testing the survival experience of one or more populations, one must not only choose an appropriate class of tests, but further an appropriate weight function. As the optimal choice depends on the true shape of the hazard ratio, one is often not capable of getting the best results with respect to a specific dataset. For the univariate case several methods were proposed to conquer this problem. However, most of the interesting datasets contain multivariate observations nowadays. In this work we propose a multivariate version of a method based on multiple constrained censored empirical likelihood where the constraints are formulated as linear functionals of the cumulative hazard functions. By considering the conditional hazards, we take the correlation between the components into account with the goal of obtaining a test that exhibits a high power irrespective of the shape of the hazard ratio under the alternative hypothesis.

## 1 INTRODUCTION

In each study testing the survival experience of one or more populations, one must choose an appropriate test. Many of those tests rely on the choice of a weight function. One can select an optimal weight function if the true shape of the hazard ratio under the alternative hypothesis is known, which is not the case in most applications.

As a result one is not capable of getting the best results with regard to a specific dataset. The choice of the weight function influences the error rates. In certain situations a wrong choice of the weight function may lead to a considerable power reduction as pointed out by Kosorok and Lin (1999) and Klein and Moeschberger (1997). In the univariate case different methods were proposed to solve the problem, for example, by Brendel, Janssen, Mayer, and Pauly (2014).

Another problem arises from the complexity of multivariate survival times. Even though in current times of massive data ascertainment, data typically contains multivariate observations, one might lack an appropriate method to model the true structure of the survival times.

For bivariate samples, many methods to analyze survival times have been proposed. The methods by Lin and Ying (1993) and Wang and Wells (1997) deal with nonparametric estimators of the bivariate survival function under univariate censoring. An earlier approach by Dabrowska (1988) can even deal with general bivariate censoring. Theoretical and computational performance comparisons of different estimators were conducted by Gill, van der Laan, and Wellner (1995) and Wang and Zafra (2009), among others. A recent paper by Huang and Zhao (2018) also deals with the bivariate survival function and uses empirical likelihood to obtain asymptotic confidence intervals.

While we mainly focus on the bivariate time‐to‐event times, our method can easily be extended to a higher dimensional case. For multivariate data with more than two response variables, methods have been proposed as well. For the multivariate case there exist less methods than in the bivariate case due to the higher complexity and technical challenges. The most common model for multivariate time‐to‐event data is the Cox model. Further, the accelerated failure time model is also often applied. A comparison of the Cox model and the accelerated failure time model, among others, was performed, for example, by Clark, Bradburn, Love, and Altman (2003b). But most multivariate methods have limitations. For example many models make parametric assumptions which may not be met, as does the Cox model which assumes proportional hazards. Other methods might lack the ability to deal correctly with dependent or correlated samples to their full extent, as their design might not pick up the correlation structure. The assumption of independence between the components is sometimes implicitly assumed, even though it is likely to be violated when dealing with different times‐to‐event of a single subject. Tests assuming independence will fail, for example, when the marginal distributions are similar for two samples, but the correlation differs. Most methods, which are primary, based on univariate test statistics can solve this problem by integrating the covariance matrices into the multivariate test statistics, as performed by Wei and Lachin (1984). An overview of multivariate survival analysis and what to consider when choosing a method for a specific dataset was given, for instance, in Clark, Bradburn, Love, and Altman (2003a, 2003c, 2003d) and Clark et al. (2003b).

The focus in this paper is motivated by the approach proposed by Bathke, Kim, and Zhou (2009). Their method is based on censored, multiply‐constrained empirical likelihood, where the constraints are formulated as linear functionals of the cumulative hazard functions. Their simulations suggested that the test performed well along with an easy computation compared to, for example, the function‐indexed weighted log‐rank tests which use extensive Monte‐Carlo simulations. The proposed method unfortunately could only deal with univariate observations.

In this paper, following the approach of Bathke et al. (2009), we propose a multivariate test statistic. In case of independent components, the approach introduced here reduces to that by Bathke et al. (2009). Test procedures designed in an univariate manner are naturally not capable of picking up correlations or dependencies. By considering the conditional hazards, we take the correlation between the components into account with the goal of obtaining a test with a high power, irrespective of the shape of the hazard ratio under the alternative hypothesis. When considering real time‐to‐event data it is unlikely that the observations within one subject are uncorrelated. It is critical to have test methods that are capable of detecting these data structures. This is why an extension of the method proposed by Bathke et al. (2009) to the multivariate case is important. Combining the conditional hazards and the constraints offers a new and well‐functioning test procedure that could be the foundation for further tests on multivariate survival times.

The paper is structured as follows. Section 2 deals with the theoretical aspects of the proposed method for the one‐sample case. Section 3 describes the results for the two‐sample case. In Section 4 we give examples on how to apply the proposed method. The simulations and an application to real data can be found in Section 5. The regularity conditions and the proofs of the main theorems are given in the Appendix.

## 2 ONE‐SAMPLE CENSORED EMPIRICAL LOG‐LIKELIHOOD WITH MULTIPLE CONSTRAINTS

We start by giving the results for the one‐sample case. We propose two methods: one assumes independence between the components, while the other one takes possible dependencies into account.

Let *X*_1_,…,*X*_*n*_ be independent, identically distributed *d*‐dimensional observations with *X*_*i*_:=(*X*_1*i*_,…,*X*_*di*_)^*T*^, distribution function *F*_*X*0_(*t*), marginal distribution functions *F*_*X*01_(*t*),…, *F*_*X*0*d*_(*t*), cumulative hazard function Λ_*X*0_(*t*), and marginal cumulative hazard functions Λ_*X*01_(*t*),…,Λ_*X*0*d*_(*t*). Due to right censoring we only observe

(1) $$T_{ji}=\min\{X_{ji},C_{ji}^{X}\}\;\text{and}\;\delta_{ji}=1\{X_{ji}\leq C_{ji}^{X}\},\;i=1,\ldots ,n,\;j=1,\ldots ,d,$$

with $C_{i}^{X}={\left(C_{1i}^{X},\ldots ,C_{di}^{X}\right)}^{T}$ the censoring times, which are assumed to be independent, identically distributed, and independent of the *X*_*i*_'s.

### 2.1 One‐sample EL with independent components

We assume in this section that the components of one observation vector are independent from each other. Based on the censored observations, the empirical log‐likelihood (EL) for component *j* pertaining to its distribution *F*_*Xj*_ is given by

(2) $$\log\;\text{EL}({\hat{F}}_{Xj})=\underset{i}{\sum }\left[\delta_{ji}\;\log\left(\Delta {\hat{F}}_{Xj}(T_{ji})\right)+(1-\delta_{ji})\log\;\left(1-{\hat{F}}_{Xj}(T_{ji})\right)\right],$$

with ${\hat{F}}_{Xj}$ the empirical marginal distribution function of component *j*=1,…,*d*. The analogue of Equation (2) based on the cumulative hazard function is given by

(3) $$\log\;\text{EL}({\hat{\Lambda }}_{Xj})=\underset{i}{\sum }[d_{ji}\log\;v_{ji}+(R_{ji}-d_{ji})\log(1-v_{ji})],$$

where

$$d_{ji}=\overset{n}{\underset{l=1}{\sum }}1\{T_{jl}=t_{ji}\}\delta_{jl}\;\text{and}\;R_{ji}=\overset{n}{\underset{l=1}{\sum }}1\{T_{jl}\geq t_{ji}\},$$

with *t*_*ji*_ denoting the ordered, distinct values of *T*_*ji*_. Here, 0<*v*_*ji*_≤1 are the discrete hazards of component *j* at time *t*_*ji*_. It is well known that the maximum of Equation (3) with respect to *v*_*ji*_ is attained at the jumps of the Nelson–Aalen estimator for each component, that is, *v*_*ji*_=*d*_*ji*_/*R*_*ji*_.

Let ***θ***:=(*θ*_11_,…,*θ*_1*k*_,…,*θ*_*d*1_,…,*θ*_*dk*_)^*T*^ be a (*k*·*d*)‐dimensional parameter defined via the components' cumulative hazard functions Λ_*X*01_,…,Λ_*X*0*d*_,

$$\theta_{jr}=\int g_{jr}(t)\;\log(1-d\Lambda_{X0j}(t)),\;r=1,\ldots ,k,\;j=1,\ldots ,d,$$

and a hypothesis testing problem

(4) $$H_{0}:\theta_{jr}=\mu_{jr}\;\forall j=1,\ldots ,d,r=1,\ldots ,k\;\text{versus}\;H_{A}:\theta_{jr}\neq \mu_{jr}\;\mathrm{for}\;\mathrm{some}\;j\;\text{and}\;r,$$

where *g*_*jr*_, *j*=1,2, *r*=1,…,*k* are nonnegative functions, and ***θ***_*j*_=(*θ*_*j*1_,…,*θ*_*jk*_)^*T*^ and ***μ***_*j*_=(*μ*_*j*1_,…,*μ*_*jk*_)^*T*^ are two vectors of constants.

The proposed constraints on the hazard *v*_*ji*_ for given functions *g*_*j*1_,…,*g*_*jk*_ and constants *μ*_*j*1_,…,*μ*_*jk*_ are given by

(5) $$\underset{i}{\sum }g_{1}(t_{ji})\;\log(1-v_{ji})=\mu_{j1},\ldots ,\;\underset{i}{\sum }g_{k}(t_{ji})\;\log(1-v_{ji})=\mu_{jk},\;j=1,2,$$

where the sum is taken over all *i* for which *v*_*ji*_≠1.

Denote the maximum empirical likelihood estimators of $\Delta {\hat{\Lambda }}_{Xj}(t_{ji})$ under constraints as $v_{ji}^{*}$, where ${\hat{\Lambda }}_{Xj}$ is the empirical cumulative hazard function. Application of the Lagrange multiplier method leads to

$$v_{ji}^{*}=v_{ji}(\lambda_{j})=\frac{d_{ji}}{R_{ji}+n\lambda_{j}^{T}G_{j}(t_{ji})},\;j=1,\ldots ,d,$$

where $G_{j}(t_{ji})={\left\{g_{j1}(t_{ji}),\ldots ,g_{jk}(t_{ji})\right\}}^{T}$, and $\lambda_{j}\in R^{k}$ is a vector of Lagrange multipliers obtained by solving the constraints for component *j*. Then the component‐wise test statistic in terms of hazards is given by

(6) $$W_{j}=-2\left[\underset{v_{ji}}{\max}\;\text{EL}(\Lambda_{Xj})\;(\text{when}\;(5)\;\text{holds})-\underset{v_{ji}}{\max}\;\text{EL}(\Lambda_{Xj})\right].$$

Theorem 1 *Define a test statistic for the complete data set by* $W:=\sum_{j=1}^{d}W_{j}$ *with W*_*j*_ *defined as in Equation (* 6 *). Then, under regularity conditions specified in the Appendix and under H*_0_ *as given in Equation (* 4 *), W is asymptotically chi‐squared distributed with k*·*d degrees of freedom for n*→∞.

Bathke et al. (2009) showed that the component‐wise test statistics *W*_*j*_ are chi‐squared distributed with *k* degrees of freedom. As we assumed independence between the components, and thus the test statistics, we obtain the desired result.

Remark 1 Although the notation suggests that the number of constraints per component is equal for all components, they may differ for the individual components, leading to different degrees of freedom. More precisely let *k*_*j*_ be the number of constraints for component *j* and define *K*=*k*_1_+…+*k*_*d*_. Then, the test statistic *W* is asymptotically chi‐squared distributed with *K* degrees of freedom under the null hypothesis.

Even though this yields a rather easy and intuitively interpretable test statistic, we encounter serious limitations. We assumed independence between the components, which may be questionable for many data sets. We tackle this problem using a more general approach in the next section.

### 2.2 General bivariate one‐sample EL

Following the approach of Dabrowska (1988) and Vonta, Nikulin, Limnios, and Huber‐Carol (2008) for bivariate survival models using conditional hazards, we derive the asymptotic distributions of the nonparametric maximum‐likelihood estimator (NPMLE) for the hazards of the bivariate survival function without constraints for the two‐dimensional case and the asymptotic distribution of the actual test statistics based on the empirical log‐likelihood function. We point out that the following sections are based on the assumption that the true underlying cumulative hazard function is continuous, even if we do not explicitly state so. Let

$$L=\{\Lambda :R^{+}\to R^{+},\text{continuous, nondecreasing},\Lambda (0)=0,\Lambda (t)\to \infty \;\text{for}\;t\to \infty \}$$

be the class of continuous univariate cumulative hazard functions on $R^{+}$. We define a joint bivariate survival function S on $R^{+}\times R^{+}$ as proposed in Dabrowska (1988) by

$$\begin{array}{ll} dS(x,y)=\; & \exp\{-\Lambda_{11}^{10}(x)-\Lambda_{11}^{01}(x)-\Lambda_{11}^{11}(x)\}d\Lambda_{11}^{10}(x)\\ & \exp\{-(\Lambda_{01}^{01}(y)-\Lambda_{01}^{01}(x)\}d\Lambda_{01}^{01}(y)\;\text{for}\;x<y,\\ dS(x,y)=\; & \exp\{-\Lambda_{11}^{10}(y)-\Lambda_{11}^{01}(y)-\Lambda_{11}^{11}(y)\}d\Lambda_{11}^{01}(y)\\ & \exp\{-(\Lambda_{10}^{10}(x)-\Lambda_{10}^{10}(y)\}d\Lambda_{10}^{10}(x)\;\text{for}\;x>y,\\ dS(x,y)=\; & \exp\{-\Lambda_{11}^{10}(x)-\Lambda_{11}^{01}(x)-\Lambda_{11}^{11}(x)\}d\Lambda_{11}^{11}(x)\;\text{for}\;x=y, \end{array}$$

where $\Lambda_{a\;b}^{c\;d}\in L$ with *a* and *b* indicating whether component 1 or 2, respectively, are event free up to *x* and *c* and *d* indicating whether an event occurred in components 1 and 2 at time point *x*. The notation is analogue to the one of the hazard rates, which are given below. This survival function *S*(*x*,*y*)=*P*(*X*≥*x*,*Y*≥*y*) is indeed a bivariate survival function. A necessary condition for the proposed method is that $\Lambda_{11}^{11}\equiv 0$ holds true, that is, the survival times of the components are not identical

The observed bivariate time is given by *T*=(*T*_1*i*_,*T*_2*i*_) and the censoring indicator is given by *δ*_*i*_=(*δ*_1*i*_,*δ*_2*i*_). Denote the hazards by

$$\lambda_{11}^{10}(t)dt=P(t\leq X_{1}\leq t+dt|X_{1}\geq t,X_{2}>t),$$

$$\lambda_{11}^{01}(t)dt=P(t\leq X_{2}\leq t+dt|X_{1}>t,X_{2}\geq t),$$

$$\lambda_{10}^{10}(t)dt=P(t\leq X_{1}\leq t+dt|X_{1}\geq t,X_{2}<t),$$

$$\lambda_{01}^{01}(t)dt=P(t\leq X_{2}\leq t+dt|X_{1}<t,X_{2}\geq t).$$

The empirical likelihood for *n* observations is the product $V=\prod_{i=1}^{n}V_{i}$ with

$$\begin{array}{ll} V_{i} & =\underset{t}{\prod }\left[{\left(1-\lambda_{11}^{10}(t)dt\right)}^{1\{T_{1i}>t\}1\{T_{2i}>t\}}\cdot {\left(\lambda_{11}^{10}(t)dt\right)}^{1\{T_{1i}=t\}1\{T_{2i}>t\}\delta_{1i}}\right.\\ & \;\cdot {\left(1-\lambda_{11}^{01}(t)dt\right)}^{1\{T_{1i}>t\}1\{T_{2i}>t\}}\cdot {\left(\lambda_{11}^{01}(t)dt\right)}^{1\{T_{1i}>t\}1\{T_{2i}=t\}\delta_{2i}}\\ & \;\cdot {\left(1-\lambda_{10}^{10}(t)dt\right)}^{1\{T_{1i}>t\}1\{T_{2i}<t\}\delta_{2i}}\cdot {\left(\lambda_{10}^{10}(t)dt\right)}^{1\{T_{1i}=t\}1\{T_{2i}<t\}\delta_{2i}\delta_{1i}}\\ & \;\left.\cdot {\left(1-\lambda_{01}^{01}(t)dt\right)}^{1\{T_{1i}<t\}1\{T_{2i}>t\}\delta_{1i}}\cdot {\left(\lambda_{01}^{01}(t)dt\right)}^{1\{T_{1i}<t\}1\{T_{2i}=t\}\delta_{2i}\delta_{1i}}\right]. \end{array}$$

For simplicity, denote the hazards with $a_{l}:=\lambda_{11}^{10}(t_{1l})dt$, $b_{l}:=\lambda_{11}^{01}(t_{2l})dt$, $c_{l}:=\lambda_{10}^{10}(t_{1l})dt$, and $d_{l}:=\lambda_{01}^{01}(t_{2l})dt$, where *t*_*jl*_ are the ordered distinct time points of jumps in component *j*. It follows that the empirical log‐likelihood *EL* is given by

(7) $$\begin{array}{ll} & \underset{t_{1l}}{\sum }\left[\log(1-a_{l})\underset{i}{\sum }(1\{T_{1i}>t_{1l}\}1\{T_{2i}>t_{1l}\})+\log(a_{l})\underset{i}{\sum }(1\{T_{1i}=t_{1l}\}1\{T_{2i}>t_{1l}\}\delta_{1i})\right]\\ & \;+\underset{t_{2l}}{\sum }\left[\log(1-b_{l})\underset{i}{\sum }(1\{T_{1i}>t_{2l}\}1\{T_{2i}>t_{2l}\})+\log(b_{l})\underset{i}{\sum }(1\{T_{1i}>t_{2l}\}1\{T_{2i}=t_{2l}\}\delta_{2i})\right]\\ & +\underset{t_{1l}}{\sum }\left[\log(1-c_{l})\underset{i}{\sum }(1\{T_{1i}>t_{1l}\}1\{T_{2i}<t_{1l}\}\delta_{2i})+\log(c_{l})\underset{i}{\sum }(1\{T_{1i}=t_{1l}\}1\{T_{2i}<t_{1l}\}\delta_{1i}\delta_{2i})\right]\\ & +\underset{t_{2l}}{\sum }\left[\log(1-d_{l})\underset{i}{\sum }(1\{T_{1i}<t_{2l}\}1\{T_{2i}>t_{2l}\}\delta_{1i})+\log(d_{l})\underset{i}{\sum }(1\{T_{1i}<t_{2l}\}1\{T_{2i}=t_{2l}\}\delta_{1i}\delta_{2i})\right], \end{array}$$

where the sums over *t*_*jl*_ are taken over those time points where *a*_*l*_, respectively *b*_*l*_, *c*_*l*_, and *d*_*l*_ are strictly greater than zero, and excluding the last time point, as the hazards would be 1. Denote the sums over ”i” by *u*_1*l*_,…,*u*_8*l*_ in the order they are given above, where *l* indicates that the sum was taken at the *l*th time point of the first component, respectively, the second component.

Lemma 1 *Maximizing the empirical log‐likelihood function with respect to a*_*l*_ *, b*_*l*_ *, c*_*l*_ *, and d*_*l*_ *, one obtains the NPMLE*

$${â}_{l}=\frac{u_{2l}}{u_{1l}+u_{2l}},\;{\hat{b}}_{l}=\frac{u_{4l}}{u_{3l}+u_{4l}},\;{ĉ}_{l}=\frac{u_{6l}}{u_{5l}+u_{6l}},\;{\hat{d}}_{l}=\frac{u_{8l}}{u_{7l}+u_{8l}}.$$

Taking the derivatives of EL with respect to *a*_*l*_, we obtain

$$-{\left(1-a_{l}\right)}^{-1}u_{1l}+a_{l}^{-1}u_{2l}.$$

The derivative is zero for ${â}_{l}=u_{2l}/(u_{1l}+u_{2l})$. For the other hazards similar calculations will lead to the given NPMLE.

When fixing the constraints we do not consider the four hazards individually, but combine them such that only one hazard per component remains for the constraints. This will lead to a reduction of the degrees of freedom, as for each constraint one degree of freedom is added to the asymptotic sampling distribution of the test statistic. Define

$$v_{1l}=P(T_{2}>t_{1l}|T_{1}\geq t_{1l})\cdot a_{l}+P(T_{2}<t_{1l}|T_{1}\geq t_{1l})\cdot c_{l}.$$

We estimate *v*_1*l*_ by *z*_1*l*_ *a*_*l*_+*z*_3*l*_ *c*_*l*_, where

$$z_{1l}=(u_{1l}+u_{2l})/(u_{1l}+u_{2l}+u_{5l}+u_{6l})=1-z_{3l}.$$

The term *v*_2*l*_ is defined similarly, namely,

$$v_{2l}=P(T_{1}>t_{2l}|T_{2}\geq t_{2l})\cdot b_{l}+P(T_{1}<t_{2l}|T_{2}\geq t_{2l})\cdot d_{l}$$

and estimated by *z*_2*l*_·*b*_*l*_+*z*_4*l*_·*d*_*l*_ with

$$z_{2l}=(u_{3l}+u_{4l})/(u_{3l}+u_{4l}+u_{7l}+u_{8l})=1-z_{4l}.$$

The constraints are then imposed directly on *v*_1*l*_ and *v*_2*l*_ and are given in the form of

$$\underset{i}{\sum }g_{jr}(t_{ji})\log(1-v_{ji})=\mu_{jr},\;j=1,2,\;r=1,\ldots ,k.$$

The function to maximize is given by

$$G_{\text{constraint}}=\text{EL}(\lambda )+\overset{2}{\underset{j=1}{\sum }}\overset{k}{\underset{r=1}{\sum }}n\lambda_{jr}[\mu_{jr}-\underset{i}{\sum }g_{jr}(t_{ji})\log(1-v_{ji})],$$

where EL(***λ***) denotes the log‐likelihood function, as given in Equation (7), without the constraints but with the modified hazards *a*_*l*_(***λ***_1_),…,*d*_*l*_(***λ***_2_) instead of the Nelson–Aalen estimators plugged in and ***λ***=(***λ***_1_,***λ***_2_)^*T*^, ***λ***_*j*_=(λ_*j*1_,…,λ_*jk*_)^*T*^, *j*=1,2. Again, the last sum ranges over the distinct time points excluding the last.

Remark 2 For survival times with absolutely continuous distribution, there is one event per time point such that either *a*_*l*_ or *c*_*l*_ will be zero. This also implies that *v*_1*l*_≤*a*_*l*_ if *c*_*l*_ is zero or *v*_1*l*_≤*c*_*l*_ if *a*_*l*_ is zero. Taking additionally into account that we exclude the last time points such that *u*_2*l*_/(*u*_1*l*_+*u*_2*l*_)≠1 and *u*_6*l*_/(*u*_5*l*_+*u*_6*l*_)≠1, we can even go as far as stating *a*_*l*_,*c*_*l*_≤0.5 and thus *v*_1*l*_≤0.5.

Remark 3 As the true NPMLE of *G*_constraint_ is rather complex to calculate, we will approximate it. Using Remark 2, we approximate $\log(1-v_{1l})$ by $z_{1l}\log(1-a_{l})+z_{3l}\log(1-c_{l})$ with an error of order $v_{1l}^{2}$, where one of the two terms is always equal to zero. In total, we approximate *G*_constraint_ by

(8) $$\begin{array}{ll} G_{\text{constraint}}^{*} & =\text{EL}(\lambda )+\overset{k}{\underset{r=1}{\sum }}n\lambda_{1r}[\mu_{1r}-\underset{i}{\sum }g_{1r}(t_{1i})(z_{1i}\;\log(1-a_{i}(\lambda_{1}))+z_{3i}\;\log(1-c_{i}(\lambda_{1})))]\\ & \;+\overset{k}{\underset{r=1}{\sum }}n\lambda_{2r}[\mu_{2r}-\underset{i}{\sum }g_{2r}(t_{2i})(z_{2i}\;\log(1-b_{i}(\lambda_{2}))+z_{4i}\;\log(1-d_{i}(\lambda_{2})))], \end{array}$$

where the sum is taken over those time points *t*_1*l*_ for which *a*_*i*_(***λ***_1_)≠1 and *c*_*i*_(***λ***_1_)≠1 and over *t*_2*i*_ for which *b*_*i*_(***λ***_1_)≠1 and *d*_*i*_(***λ***_1_)≠1.

Remark 4 For noncontinuous distributions *v*_*jl*_ can be close to zero just like in the continuous case. It may, however, occur in such cases that we obtain high hazard rates at some of the jump points. Thus the approximation as described in Remark 3 would not hold in general. Therefore, we will exclude noncontinuous distributions in the following.

Remark 5 The approximation is a true equation if the components are independent, that is, the conditional hazards can be replaced by the regular hazards for the individual components. In this case, the conditional hazard model simplifies to the sum of the univariate models and thus we obtain the same results as in Section 2.1.

Remark 6 Instead of approximating *G*_constraint_ one can also simply define the parameter *θ* under the null hypothesis to be the weighted sum of the integrals with respect to the conditional component‐wise cumulative hazard function instead of the component‐wise cumulative hazard function. That is, define

$$\begin{array}{ll} \theta_{1r} & =\int P(T_{2}>t|T_{1}>t)g_{r}(t)\;\log(1-d\Lambda_{X01|2>})\\ & \;+\int P(T_{2}<t|T_{1}>t)g_{r}(t)\;\log(1-d\Lambda_{X01|2<}), \end{array}$$

for the constraints on the first component, with Λ_*X*01|2>_ and Λ_*X*01|2<_ the conditional component‐wise cumulative hazard functions. Note that this approach might be more precise. However, in order to test the hypothesis one must make assumptions on the conditional probabilities, which are usually unknown and difficult to estimate in advance in the absence of a pilot study.

Theorem 2 *The NPMLE of G*_constraints_ *, as given in Equation (* 8 *), are given by*

$$\begin{array}{ll} {â}_{l}^{*} & =a_{l}(\lambda_{1})=\frac{u_{2l}}{u_{1l}+u_{2l}+z_{1l}n\lambda_{1}^{T}G_{1}(t_{1l})},\;{\hat{b}}_{l}^{*}=b_{l}(\lambda_{2})=\frac{u_{4l}}{u_{3l}+u_{4l}+z_{2l}n\lambda_{2}^{T}G_{2}(t_{2l})},\\ {ĉ}_{l}^{*} & =c_{l}(\lambda_{1})=\frac{u_{6l}}{u_{5l}+u_{6l}+z_{3l}n\lambda_{1}^{T}G_{1}(t_{1l})},\;{\hat{d}}_{l}^{*}=d_{l}(\lambda_{2})=\frac{u_{8l}}{u_{7l}+u_{8l}+z_{4l}n\lambda_{2}^{T}G_{2}(t_{2l})}, \end{array}$$

*where* ***λ***_1_ *and* ***λ***_2_ *are obtained as the solution of the following* 2*k equations*:

(9) $$\underset{i}{\sum }g_{11}(t_{1i})\log(1-v_{1i}(\lambda_{1}))=\mu_{11},\ldots ,\;\underset{i}{\sum }g_{2k}(t_{2i})\;\log(1-v_{2i}(\lambda_{2}))=\mu_{2k},$$

*with v*_1*i*_(***λ***_1_):=*z*_1*l*_ *a*_*l*_(***λ***_1_)+*z*_3*l*_ *c*_*l*_(***λ***_1_) *and v*_2*i*_(***λ***_2_):=*z*_2*l*_ *b*_*l*_(***λ***_2_)+*z*_4*l*_ *d*_*l*_(***λ***_2_).

*Further* ***G***_1_(*t*_1*l*_):=(*g*_11_(*t*_1*l*_),…,*g*_1*k*_(*t*_1*l*_))^*T*^ *and* ***G***_2_(*t*_2*l*_):=(*g*_21_(*t*_2*l*_),…,*g*_2*k*_(*t*_2*l*_))^*T*^.

Deriving the approximation of *G*_constraint_ with respect to the λs simply results in the constraints. For instance, the derivatives of *G*_constraint_ with respect to (w.r.t.) *a*_*l*_ leads to

$$\frac{u_{2l}}{a_{l}}-\frac{u_{1l}}{1-a_{l}}-n\lambda_{1}^{T}G_{1}(t_{1l})\cdot \frac{z_{1l}}{1-a_{l}}.$$

Equalizing the previous expression to zero and solving for *a*_*l*_ leads to the NPMLE

$${â}_{l}^{*}=\frac{u_{2l}}{u_{1l}+u_{2l}+z_{1l}n\lambda_{1}^{T}G_{1}(t_{1l})}.$$

The remaining derivations follow analogously.

Let the test statistic in terms of hazards be given by

$$W=-2\{\max G_{\text{constraint}}-\max\;\text{EL}(\Lambda_{X})\},$$

where EL(Λ_*X*_) is empirical log‐likelihood as given in Equation (7).

Theorem 3 *Suppose that the null hypothesis H*_0_ *, as defined in Equation (* 4 *), holds for nonnegative, random functions g*_*jr*_(*t*) *that are predictable w.r.t. the filtration* $F_{t}=\sigma \{T_{1i}1\{T_{1i}\leq t\};\delta_{1i}1\{T_{1i}\leq t\};T_{2i}1\{T_{2i}\leq t\};\delta_{2i}1\{T_{2i}\leq t\};i=1,\ldots ,n\}$ *. Then, under regularity conditions specified in the Appendix, the test statistic W has asymptotically a chi‐squared distribution with* 2*k degrees of freedom, where k is the number of constraints per dimension*.

As in the case of independent components we can vary the number of constraints per component, such that for every component a fitting number of constraints can be chosen.

### 2.3 General *d*‐dimensional one‐sample EL

As mentioned earlier, the presented approach can be extended to any arbitrary number of components. However, the notation in a case beyond the bivariate model gets rather complex. In general, one considers all conditional hazards that could possibly occur. For the three‐dimensional case this would be

$$\begin{array}{ll} \lambda_{111}^{100}(t)dt & =P(t\leq X_{1}\leq t+dt|X_{1}\geq t,X_{2}>t,X_{3}>t)=:a_{1}(t),\\ \lambda_{110}^{100}(t)dt & =P(t\leq X_{1}\leq t+dt|X_{1}\geq t,X_{2}>t,X_{3}<t)=:a_{2}(t),\\ \lambda_{101}^{100}(t)dt & =P(t\leq X_{1}\leq t+dt|X_{1}\geq t,X_{2}<t,X_{3}>t)=:a_{3}(t),\\ \lambda_{100}^{100}(t)dt & =P(t\leq X_{1}\leq t+dt|X_{1}\geq t,X_{2}<t,X_{3}<t)=:a_{4}(t),\\ \lambda_{111}^{010}(t)dt & =P(t\leq X_{2}\leq t+dt|X_{1}>t,X_{2}\geq t,X_{3}>t)=:b_{1}(t),\\ \lambda_{111}^{001}(t)dt & =P(t\leq X_{3}\leq t+dt|X_{1}>t,X_{2}>t,X_{3}\geq t)=:c_{1}(t), \end{array}$$

and so forth. In general, the number of conditional hazards in the *d*‐dimensional case is given by *d*·2^(*d*−1)^. The remaining derivations follow analogously to the bivariate case. Note that the complete hazard is then summed up over all conditions, which are four for the three‐dimensional case. Explicitly this means for the hazard of the first component that

$$v_{1l}=P(T_{2}>t_{1l},T_{3}>t_{1l}|T_{1}\geq t_{1l})\cdot a_{1l}+\ldots +P(T_{2}<t_{1l},T_{3}<t_{1l}|T_{1}\geq t_{1l})\cdot a_{4l}.$$

Denote the log‐likelihood function, similar to Equation (7), by EL and consider constraints of the form

$$\underset{i}{\sum }g_{jr}(t_{ji})\log(1-v_{ji})=\mu_{jr},\;j=1,\ldots ,d,\;r=1,\ldots ,k_{j}.$$

Then the function to maximize is given by

$$G_{\text{constraint}}(\mu )=\text{EL}(\lambda )+\overset{d}{\underset{j=1}{\sum }}\overset{k_{j}}{\underset{r=1}{\sum }}n\lambda_{jr}\left[\mu_{jr}-\underset{i}{\sum }g_{jr}(t_{ji})\log(1-v_{ji})\right].$$

Theorem 4 *Suppose that the null hypothesis as defined in Equation (* 4 *) holds for nonnegative, random functions g*_*jr*_(*t*)*. Under the regularity conditions specified in the Appendix, the test statistic defined by*

$$W=W(\mu ):=-2\{\max\;G_{\text{constraint}}(\mu )-\max\;\mathrm{EL}(\Lambda_{X})\}$$

*has asymptotically a chi‐squared distribution with* ${\;\sum }_{j=1}^{d}k_{j}$ *degrees of freedom with k*_*j*_ *the number of constraints in dimension j*.

Remark 7 Although the multiple testing procedure is only applied to hypothesis testing here, it can be used for other forms of inference as well. For example it could be used to obtain confidence regions for a collection of the population quantiles. A straightforward way to construct confidence regions for the true $\theta_{0}={\left(\theta_{011},\ldots ,\theta_{01k_{1}},\ldots ,\theta_{0d1},\ldots ,\theta_{0dk_{d}}\right)}^{T}$ works as follows. Define the confidence region to contain all $\mu ={\left(\mu_{11},\ldots ,\mu_{dk_{d}}\right)}^{T}$ for which the test statistic *W*(***μ***) as given in Theorem 4 is smaller than the 1−*α*‐quantile of the chi‐squared distribution with $\sum_{j=1}^{d}k_{j}$ degrees of freedom.

## 3 TWO‐SAMPLE CENSORED EL WITH MULTIPLE CONSTRAINTS

As it is often of interest to not only test certain aspects of one sample but further to compare it with a second sample, we expand the model to a two‐sample censored EL. Now, additionally to our partly censored sample of observations *X*_1_,…,*X*_*n*_, we have a second *d*‐dimensional i.i.d. sample *Y*_1_,…,*Y*_*m*_ with distribution function *F*_*Y*0_(*t*), marginal distribution functions *F*_*Y*01_(*t*), …, *F*_*Y*0*d*_(*t*), cumulative hazard function Λ_*Y*0_(*t*), and marginal cumulative hazard functions Λ_*Y*01_(*t*), …, Λ_*Y*0*d*_(*t*). Assume that the *Y*_*i*_s, *i*=1,…,*m*, are independent of the *X*_*j*_s, *j*=1,…,*n*. Again, the sample may be censored, such that we only observe $S_{ji}=\min(Y_{ji},C_{ji}^{Y})$ and $\tau_{ji}=1\{Y_{ji}\leq S_{ji}\}$, where the $C_{i}^{Y}=(C_{1i}^{Y},\ldots ,C_{di}^{Y})$ denote the censoring times for the second sample, which shall be independent from the *Y*_*i*_. Denote the ordered, distinct values of the *S*_*ji*_ as *s*_*jl*_.

Note that we do not require independence between the individual components for this method.

### 3.1 Bivariate two‐sample EL

The EL function based on the two samples is given by

(10) $$\log\;\text{EL}(\Lambda_{X}+\Lambda_{Y})={\text{EL}}_{X}+{\text{EL}}_{Y},$$

where EL_*X*_ corresponds to the EL function of the observations *X*, as given in Equation (7), and EL_*Y*_ corresponds to the EL function of the observations *Y*, analogously to EL_*X*_. More explicitly, EL_*Y*_ is given by

$$\begin{array}{ll} & \underset{s_{1l}}{\sum }\left[\log(1-\alpha_{l})\underset{i}{\sum }(1\{S_{1i}>s_{1l}\}1\{S_{2i}>s_{1l}\})+\log(\alpha_{l})\underset{i}{\sum }(1\{S_{1i}=s_{1l}\}1\{S_{2i}>s_{1l}\}\tau_{1i})\right]\\ & \underset{s_{2l}}{\sum }\left[\log(1-\beta_{l})\underset{i}{\sum }(1\{S_{1i}>s_{2l}\}1\{S_{2i}>s_{2l}\})+\log(\beta_{l})\underset{i}{\sum }(1\{S_{1i}>s_{2l}\}1\{S_{2i}=s_{2l}\}\tau_{2i})\right]\\ & \underset{s_{1l}}{\sum }\left[\log(1-\gamma_{l})\underset{i}{\sum }(1\{S_{1i}>s_{1l}\}1\{S_{2i}<s_{1l}\}\tau_{2i})+\log(\gamma_{l})\underset{i}{\sum }(1\{S_{1i}=s_{1l}\}1\{S_{2i}<s_{1l}\}\tau_{1i}\tau_{2i})\right]\\ & \underset{s_{2l}}{\sum }\left[\log(1-\varepsilon_{l})\underset{i}{\sum }(1\{S_{1i}<s_{2l}\}1\{S_{2i}>s_{2l}\}\tau_{1i})+\log(\varepsilon_{l})\underset{i}{\sum }(1\{S_{1i}<s_{2l}\}1\{S_{2i}=s_{2l}\}\tau_{1i}\tau_{2i})\right], \end{array}$$

where the sums over *s*_*jl*_ are only taken over those time points where *α*_*l*_, respectively *β*_*l*_, *γ*_*l*_, and *ε*_*l*_ are truly greater than zero, and excluding the last time points, as there the hazards would be 1. Again, we will denote the eight sums over *i* with ν_1*l*_,…,ν_8*l*_, in the same order as they appear above.

Lemma 2 *Maximizing the log‐likelihood function w.r.t*. *a*_*l*_,…,*ε*_*l*_ *, one obtains the NPMLE*

$$\begin{array}{ll} {â}_{l} & =\frac{u_{2l}}{u_{1l}+u_{2l}},\;{\hat{b}}_{l}=\frac{u_{4l}}{u_{3l}+u_{4l}},\;{ĉ}_{l}=\frac{u_{6l}}{u_{5l}+u_{6l}},\;{\hat{d}}_{l}=\frac{u_{8l}}{u_{7l}+u_{8l}},\\ {\hat{\alpha }}_{l} & =\frac{\nu_{2l}}{v_{1l}+\nu_{2l}},\;{\hat{\beta }}_{l}=\frac{\nu_{4l}}{\nu_{3l}+\nu_{4l}},\;{\hat{\gamma }}_{l}=\frac{\nu_{6l}}{\nu_{5l}+\nu_{6l}},\;{\hat{\varepsilon }}_{l}=\frac{\nu_{8l}}{\nu_{7l}+\nu_{8l}}, \end{array}$$

*where l indicates the time point of the jump*.

Let us now consider a hypothesis testing problem for a 2*k*‐dimensional parameter ***θ***=(*θ*_11_,…,*θ*_1*k*_,*θ*_21_,…,*θ*_2*k*_)^*T*^ w.r.t. the cumulative hazard functions Λ_*X*_ and Λ_*Y*_ such that

(11) $$H_{0}:\theta =\mu \;\text{vs.}\;H_{A}:\theta \neq \mu ,$$

where

$$\theta_{jr}=\int g_{jr}(t)\;\log(1-d\Lambda_{X0j}(t))-\int h_{jr}(t)\;\log(1-d\Lambda_{Y0j}(t)),\;r=1,\ldots ,k,\;j=1,2,$$

for some given functions *g*_*jr*_(*t*) and *h*_*jr*_(*t*). Then, the constraints imposed on *v*_*ji*_ and *w*_*ji*_ shall be given by

(12) $$\mu_{jr}=\underset{i}{\sum }{\;g}_{jr}(t_{ji})\;\log(1-v_{ji})-\underset{i}{\sum }h_{jr}(s_{ji})\;\log(1-w_{ji}),$$

with *j*=1,2, *r*=1,…,*k*, and where the sum is taken over all distinct time points in each sample, excluding the last value as the hazard would be 1 at that time point. The *w*_*ji*_s are defined analogously to *v*_*ji*_s,

$$w_{1i}(\lambda_{1}):=\zeta_{1l}\alpha_{l}(\lambda_{1})+\zeta_{3l}\gamma_{l}(\lambda_{1})\;\text{and}\;w_{2i}(\lambda_{2}):=\zeta_{2l}\beta_{l}(\lambda_{2})+\zeta_{4l}\varepsilon_{l}(\lambda_{2}).$$

Application of the Lagrange multiplier method on the likelihood with constraints as given in Equation (12) shows that the NPMLE are given through

(13) $$\begin{array}{ll} {â}_{l}^{*}=\frac{u_{2l}}{u_{1l}+u_{2l}+z_{1l}n^{*}\lambda_{1}^{T}G_{1}(t_{1l})},\;{\hat{b}}_{l}^{*}=\frac{u_{4l}}{u_{3l}+u_{4l}+z_{2l}n^{*}\lambda_{2}^{T}G_{2}(t_{2l})}, & \\ {ĉ}_{l}^{*}=\frac{u_{6l}}{u_{5l}+u_{6l}+z_{3l}n^{*}\lambda_{1}^{T}G_{1}(t_{1l})},\;{\hat{d}}_{l}^{*}=\frac{u_{8l}}{u_{7l}+u_{8l}+z_{4l}n^{*}\lambda_{2}^{T}G_{2}(t_{2l})}\;, & \\ {\hat{\alpha }}_{l}^{*}=\frac{\nu_{2l}}{\nu_{1l}+\nu_{2l}-\zeta_{1l}n^{*}\lambda_{1}^{T}H_{1}(s_{1l})},\;{\hat{\beta }}_{l}^{*}=\frac{\nu_{4l}}{\nu_{3l}+\nu_{4l}-\zeta_{2l}n^{*}\lambda_{2}^{T}H_{2}(s_{2l})}, & \\ {\hat{\gamma }}_{l}^{*}=\frac{\nu_{6l}}{\nu_{5l}+\nu_{6l}-\zeta_{3l}n^{*}\lambda_{1}^{T}H_{1}(s_{1l})},\;{\hat{\varepsilon }}_{l}^{*}=\frac{\nu_{8l}}{\nu_{7l}+\nu_{8l}-\zeta_{4l}n^{*}\lambda_{2}^{T}H_{2}(s_{2l})}, & \end{array}$$

where ***G***_1_(*t*_1*l*_), ***G***_2_(*t*_2*l*_), ***H***_1_(*s*_1*l*_), and ***H***_2_(*s*_2*l*_) denote the vectors of constraints imposed on the hazards at the time point *t*_*jl*_, respectively *s*_*jl*_, and *n* ^∗^ is defined as min(n,m).

Let *G*_constraint_ be given by

$$\begin{array}{ll} G_{\text{constraint}} & ={\text{EL}}_{X}(\lambda )+{\text{EL}}_{Y}(\lambda )\\ & \;+\overset{2}{\underset{j=1}{\sum }}\overset{k}{\underset{r=1}{\sum }}n^{*}\lambda_{jr}\left[\mu_{jr}-\left(\underset{i}{\sum }g_{jr}(t_{ji})\;\log(1-v_{ji})-\underset{i}{\sum }h_{jr}(s_{ji})\;\log(1-w_{ji})\right)\right], \end{array}$$

where EL_*X*_(***λ***) and EL_*Y*_(***λ***) denote the log‐likelihood function without constraints based on the modified hazards *a*_*l*_(***λ***),…,*ε*_*l*_(***λ***) instead of the Nelson–Aalen estimators.

The two‐sample test statistic is given by

(14) $$W^{*}:=-2\left(\max{\;G}_{\text{constraint}}-\max({\text{EL}}_{X}+{\text{EL}}_{Y})\right).$$

Theorem 5 *Suppose that the null hypothesis H*_0_:*θ*_*jr*_=*μ*_*jr*_ *holds for all j*=1,2 *and r*=1,…,*k, that is,*

$$\mu_{jr}=\int g_{jr}(t)\;\log(1-d\Lambda_{X0j}(t))-\int h_{jr}(t)\;\log(1-d\Lambda_{Y0j}(t)),$$

*for nonnegative random functions g*_*jr*_(*t*) *and h*_*jr*_(*t*) *that are predictable w.r.t. the filtration* $F_{t}$ *specified in Appendix. Then, under further conditions specified in the regularity specification for the two‐sample case, as n* ^∗^→∞ *and n*/*m*→*c*∈(0,∞)*, W* ^∗^ *, as given in Equation (* 14 *), has asymptotically a chi‐squared distribution with* 2*k degrees of freedom*.

Again the number of constraints can be fitted individually to match each component. Needless to mention, the number of constraints for the two samples must match.

### 3.2 General *d*‐dimensional two‐sample EL

As in the one‐sample case, the proposed method works for any arbitrary number of components. Due to complexity of notation we only sketch the idea of the general *d*‐dimensional two‐sample test.

Let EL(λ) be the sum of the individual EL functions in each component with again the modified hazards instead of the Nelson–Aalen estimators. Further let EL be the sum of the individual EL functions in each component with the Nelson–Aalen estimators for the hazards. The function to maximize under the constraints, *G*_constraint_, is given by

$$G_{\text{constraint}}=\text{EL}(\lambda )+\overset{d}{\underset{j=1}{\sum }}\overset{k_{j}}{\underset{r=1}{\sum }}n^{*}\lambda_{jr}\left[\mu_{jr}-\left(\underset{i}{\sum }g_{jr}(t_{ji})\;\log(1-v_{ji})-\underset{i}{\sum }h_{jr}(s_{ji})\;\log(1-w_{ji})\right)\right].$$

Theorem 6 *Suppose that the null hypothesis H*_0_:*θ*_*jr*_=*μ*_*jr*_ *holds for all j*=1,…,*d and r*=1,…,*k*_*j*_ *, that is,*

$$\mu_{jr}=\int g_{jr}(t)\;\log(1-d\Lambda_{X0j}(t))-\int h_{jr}(t)\;\log(1-d\Lambda_{Y0j}(t)),$$

*for nonnegative random functions g*_*jr*_(*t*) *and h*_*jr*_(*t*) *that are predictable w.r.t. the filtration* $F_{t}$ *specified in Appendix. Then under further conditions specified in the regularity specification for the two‐samplecase, as n* ^∗^→∞ *and n*/*m*→*c*∈(0,∞)*,*

$$W^{*}=-2\{\max{\;G}_{\text{constraint}}-\max\;EL\},$$

*has asymptotically a chi‐squared distribution with* $\sum_{j=1}^{d}k_{j}$ *degrees of freedom*.

## 4 APPLICATIONS

In this section we discuss different applications of Theorem 5. The constraints proposed in the following are based on random yet predictable constraint functions *g*_*jr*_ and *h*_*jr*_.

One application of Theorem 5 is based on constraints obtained by combining the log‐rank test with the Gehan test. We consider a two‐sample problem with *H*_0_:Λ_*X*_(*t*)=Λ_*Y*_(*t*) for all *t*≥0 versus *H*_1_:Λ_*X*_(*t*)≠Λ_*Y*_(*t*) for some *t*. Specifically the test statistics of the individual tests can be given in the form of

$$\underset{i}{\sum }h_{j}^{*}(t_{ji},\rho ,\gamma )\log(1-v_{ji})-\underset{l}{\sum }h_{j}^{*}(s_{jl},\rho ,\gamma )\log(1-w_{jl})=\mu_{j},\;j=1,2,$$

where

$$h_{j}^{*}(u,\rho ,\gamma )={\left(\frac{n+m}{nm}\right)}^{1/2}W_{j}{\left(u\right)}^{\rho }{\left(1-W_{j}(u)\right)}^{\gamma }\frac{R_{j1}(u)R_{j2}(u)}{R_{j1}(u)+R_{j2}(u)},\;\text{for}\;\rho ,\gamma \geq 0,$$

as well as $R_{j1}(u)=\sum 1\{T_{ji}\geq u\}$ and $R_{j2}(u)=\sum 1\{S_{ji}\geq u\}$.

If *W*_*j*_(*u*) equals (*R*_*j*1_(*u*)+*R*_*j*2_(*u*))/(*n*+*m*) then (*ρ*,*γ*)=(0,0) corresponds to the log‐rank statistic and (*ρ*,*γ*)=(1,0) corresponds to the Gehan–Wilcoxon statistic. Note that $h_{j}^{*}(u,\rho ,\gamma )$, *j*=1,2, are indeed predictable functions.

For the proposed method, we used

$$\underset{i}{\sum }h_{j}^{*}(t_{ji},\rho ,\gamma )\log(1-v_{ji})-\underset{l}{\sum }h_{j}^{*}(s_{jl},\rho ,\gamma )\log(1-w_{jl})=0,$$

as constraints for the two combinations of (*ρ*,*γ*) mentioned above. More specifically, we applied the constraint functions

(15) $$g_{j1}(u)=h_{j1}(u)=\left(\frac{n+m}{nm}\right)\frac{R_{j1}(u)R_{j2}(u)}{R_{j1}(u)+R_{j2}(u)},\;j=1,2,$$

namely the log‐rank statistic, and

(16) $$g_{j2}(u)=h_{j2}(u)=\left(\frac{n+m}{nm}\right)\frac{R_{j1}(u)+R_{j2}(u)}{n+m},\;j=1,2,$$

the Gehan–Wilcoxon statistic, to each component leading to four degrees of freedom.

A comparison of the combined test based on these constraints with the two individual tests was performed in a simulation study. It corresponds to Simulation 4 in Section 5.

Further, it might be of interest to test whether two distributions which have identical marginals are identically distributed also in the multivariate sense. In practice, it is possible that two datasets differ only in the correlation structure, which can easily go undetected. To this end, one may apply functions of the form

(17) $$f(t)=1\{\exists j\;s.t.\;T_{2j}=t,\delta_{2j}=1\;\mathrm{and}\;T_{1j}>t\}g(t),$$

with *g*(*t*) a nonnegative predictable function. Applying, for instance, five constraints we can use four to focus on testing the two datasets for equal marginals. Two may be used for the first component, where one of them puts weight on early events, and the other one on late events, and the same for the second component. The last constraint can be used to control whether the correlation within each of the two datasets is similar, and it can be applied to either component. If it is of the form Equation (17) it should be applied to the second component. As one can see in Section 5 this leads to a high rejection level if the correlation structure is not the same, even if the samples are drawn from identical marginal distributions.

Instead of using random, predictable functions, one can also apply various deterministic functions as constraints. Those can be chosen such that the test focuses on the part of the data where differences are to be expected or are considered as critical time frames. For instance, if one is only interested in detecting differences at the beginning of the study, constraints of the form $g_{1}(t)=\exp\{-t\}$ or $g_{2}(t)=1\{t\leq t^{*}\},\;t^{*}\in R^{+}$ can be applied. While *g*_1_(*t*) assigns greater weight to earlier time points and lesser weight to later ones, *g*_2_(*t*) weights all time points up to a prespecified time point *t* ^∗^ equally and does not consider later time points at all. On the other hand, if one is interested in differences at later time points, one can apply constraints of the form $g_{1}(t)=1\{t\geq t^{*}\},\;t^{*}\in R^{+}$ or *g*_2_(*t*)=*log*(*t*+1), among others. Obviously, it is possible to combine two constraints so that one detects differences at early time points and one at later time points. Constraints of this form were used in the first simulation as given in Section 5.

## 5 SIMULATIONS AND DATA EXAMPLE

We provide simulation results that confirm the chi‐squared limiting distributions of *W* and *W* ^∗^ and illustrate the small sample performance. Further, we ran some simulations to show how dependency and correlation structures influence the results. We then compared the performance of our test to various others. A real data example is provided to illustrate the proposed method.

**Figure 1:**
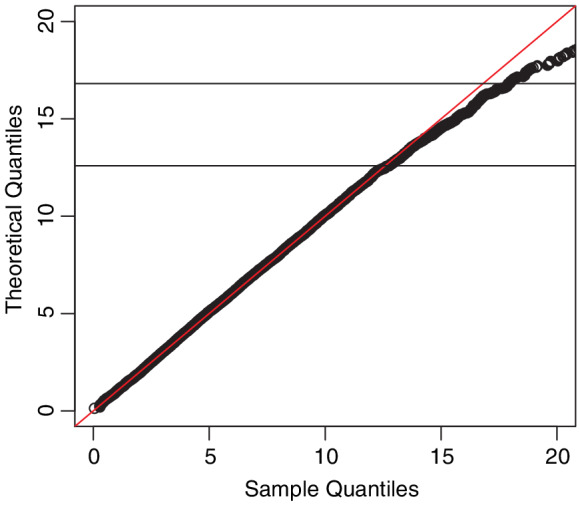
Q‐Q plot of the empirical quantiles versus $X_{6}^{2}$ percentiles for sample size 200 in the one sample case, corresponding to the theoretical result in Theorem 3 [Colour figure can be viewed at wileyonlinelibrary.com]

All computations were performed using R (R version 3.3.2, R Core Team, 2017).

Simulation 1 (one‐sample case) 1 This simulation illustrates the empirical distribution of *W* when the constraints are given by the functions

$$g_{1}(t)=\exp\{-t\},\;g_{2}(t)=0.5t1\{t\leq 1\},\;g_{3}(t)=1\{0.5<t<1.5\}.$$

All constraints were applied to both components. We used the following distributions to generate the bivariate random variables:

$$X_{1}∼\exp(0.9),\;X_{2}∼\exp(0.6),\;C_{1}∼\exp(0.5),\;C_{2}∼\exp(0.3).$$

The actual observed data was then created analogously to Equation (1). Censoring was roughly one‐third in each of the components. The observations were uncensored in both components in 42*%* of the cases.

The Q‐Q plot (Figure 1) is based on 10,000 runs and a sample size of 200. The resulting empirical distribution of *W* agrees well with the theoretically derived $X_{6}^{2}$‐distribution.

Simulation 2 (two‐sample case) 1 In this simulation we illustrate the empirical distribution of *W* ^∗^ for the two‐sample case. The constraints are given by the functions

$$\begin{array}{ll} g_{1}(t) & =\exp\{-t\},\;g_{2}(t)=0.5t1\{t\leq 1\},\\ g_{3}(t) & =1\{t<=0.9\},\;g_{4}(t)=2\exp\{-t\}1\{t>0.5\}. \end{array}$$

The functions *g*_1_ and *g*_2_ were applied to the first component and the remaining two to the second component. The two samples were drawn from the following marginal distributions:

$$X_{1},Y_{1}∼\exp(0.9),\;X_{2},Y_{2}∼\exp(0.6),\;C_{1}^{X},C_{1}^{Y}∼\exp(0.5),\;C_{2}^{X},C_{2}^{Y}∼\exp(0.3).$$

The simulated actual observations after censoring were again created according to Equation (1). The amount of censoring was 36*%* for the first component and 73*%* for the second component.

**Figure 2:**
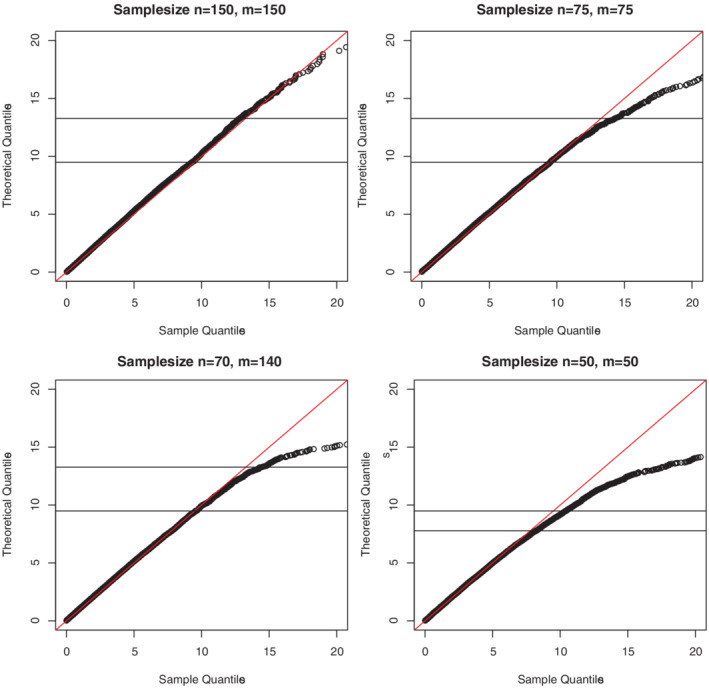
Q‐Q plot of the empirical quantiles versus $X_{4}^{2}$ percentiles for different sample sizes in the two‐sample case, corresponding to the result in Theorem 5 [Colour figure can be viewed at wileyonlinelibrary.com]

The four Q‐Q plots in Figure 2 are based on 10,000 runs and show different combinations of sample sizes. The resulting empirical distribution of *W* generally agreed well with the theoretically derived $X_{4}^{2}$‐distribution. However, in the case of *m*=*n*=50 it only agreed well with the theoretical distribution up to the 90*%* quantile.

Table 1 shows the Type I errors at various significance levels and different sample sizes. The proposed combined test attained the Type I error quite well at the nominal levels for sample sizes of *n*,*m*≥70.

**Table 1:** Estimated Type I errors at various significance levels *α* and different sample sizes for two samples, corresponding to the theoretical results in Theorem 5

				*α*		
*n*	*m*	0.01	0.05	0.1	0.15	0.2
50	50	0.0210	0.0667	0.1140	0.1593	0.2060
75	75	0.0128	0.0505	0.0945	0.1416	0.1886
150	150	0.0082	0.0468	0.0884	0.1302	0.1769
70	140	0.0132	0.0517	0.1007	0.1463	0.1925

Simulation 3 (Dependency/correlation structure) 1 The previous simulations were performed with random variables which had independent components. The following simulations show the influence of high dependency on the test statistic and the consequences of identically distributed components but with different correlation structures in the two datasets. First, we considered bivariate random variables which were identically distributed in both samples. For the first component we drew samples according to

$$X_{1},Y_{1}∼\exp(0.9),\;C_{1}^{X},C_{1}^{Y}∼\exp(0.3).$$

The second components were obtained by *X*_2_=0.65*X*_1_+0.35*X* and *Y*_2_=0.65*Y*_1_+0.35*Y*, with X,Y∼exp(0.6). Censoring was still considered to be i.i.d., such that $C_{2}^{X},C_{2}^{Y}∼\exp(0.3)$. The censoring accumulated to 25*%* in the first component and 30*%* in the second. Overall 55*%* of the observations could be observed completely. For each dataset we simulated 200 observations. The constraints were given by the functions

$$\begin{array}{ll} g_{1}(t) & =\exp\{-t\},\;g_{2}(t)=0.5t1\{t\leq 1\},\\ g_{3}(t) & =1\{t<=0.9\},\;g_{4}(t)=2\exp\{-t\}1\{t>0.5\}. \end{array}$$

Here, *g*_1_ and *g*_2_ were applied to the first component, the remaining two to the second component.

**Figure 3:**
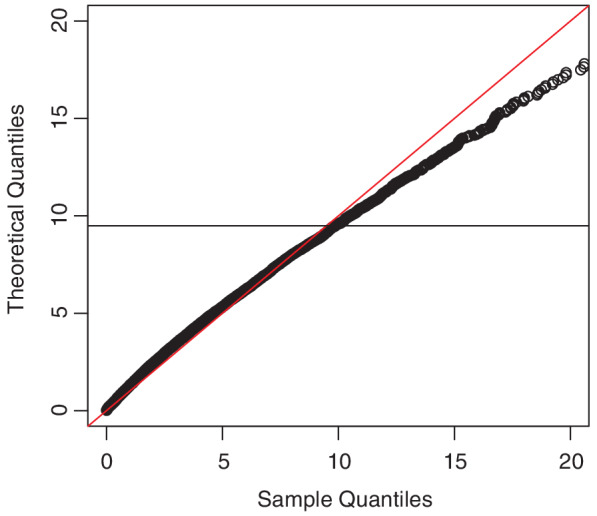
Q‐Q plot of the empirical quantiles versus $X_{4}^{2}$ percentiles for sample size 200 in the two‐sample case, corresponding to the theoretical result in Theorem 5 [Colour figure can be viewed at wileyonlinelibrary.com]

The Q‐Q plot (Figure 3) is based on 10,000 runs. The resulting empirical distribution of *W* ^∗^ agreed with the theoretically derived $X_{4}^{2}$‐distribution up to the 90*%* quantile. For even stronger dependencies, higher sample sizes are needed to obtain similar empirical results. Yet we can conclude that with a sufficient number of observations, the proposed method can deal with dependencies.

Correlation between the components may also be of interest in the case of multivariate data. Extreme cases of testing two samples for equality could be given by having identical marginals but different correlation structure. A test should reject the null hypothesis of identical joint survival distributions in such cases. Both samples were simulated by

$$X_{1},Y_{1}∼\exp(0.9),\;X_{2},Y_{2}∼\exp(0.9),\;C_{1}^{X},C_{1}^{Y}∼\exp(0.2),\;C_{2}^{X},C_{2}^{Y}∼\exp(0.2).$$

To obtain a different correlation structure, the samples of *X*_1_ and of *X*_2_ were sorted. The correlation of the true observations of *X* was given by .99 and for *Y* by 0. The actual observed data of the *X*‐sample, *T*_*X*_, was only correlated by .68. The correlation for the actual observed data for the *Y*‐sample remained the same. The constraints were given by

$$g_{1}(t)=\exp\{-t\},\;g_{2}(t)=1\{t\leq 1.5\},\;g_{3}(t)=21\{0.5<t<1.5\},$$

and *g*_4_ as defined in Equation (17) with *g*(*t*)=1. Again, the first two constraints were imposed on component 1 and the other two on component 2. The simulated power was 81.79*%* indicating that the proposed method indeed is capable of detecting differences in the correlation structure. As one can see in Simulation 4, other methods have limited capability to detect such differences.

Simulation 4 (Comparison with other methods) 1 We compare the small and moderate sample size behaviors of the proposed combined tests, as described in the first application in Section 4, to two tests from the *G* ^*ρ*,*γ*^ family of Harrington and Fleming (1982). As the constraints correspond to the weight functions of the log‐rank statistic and the Gehan statistic, we compared the combined constraints to the individual tests. For all three tests, we estimated the Type I errors for one null hypothesis and the power for five different alternatives. The size of the simulation was 10,000 for the null model and 5,000 for each of the alternative models A–E, as defined in Table 2.

The sample size for the small sample size setting was given by 50 *X*‐observations and 50 *Y*‐observations. For the moderate sample size setting we had 150 *X*‐observations and 150 *Y*‐observations. The five different survival configurations for the power analyses A–E are described as follows. A corresponds to identical marginals but different correlation structure, B to one component identically distributed and one different, C to crossing hazards, D to ordered hazards with early differences, and E to ordered hazards with late differences. Samples were generated both without censoring and with exponentially distributed censoring with parameter λ_1_=0.5 for the first component and λ_2_=0.3 for the second component such that censoring amounted to 66*%* and 64*%* in the two components in case of the null model. Further information on the modeling can be found in Table 2.

For alternative A, an additional constraint was added to the second component, which was given by the constraint function in Equation (17) with *g*(*t*)=1. The other constraints correspond to Equations (15) and (16), which are random predictable functions w.r.t. the filtration we specified in the Appendix B1. Both constraints were applied to each component.

**Table 2:** Hazard functions for the configurations used in the power studies (Simulation 4)

Configuration	Hazard functions	*df*
A	λ_*X*1_(*t*)=λ_*X*2_(*t*)=λ_*Y*1_(*t*)=λ_*Y*2_(*t*)=0.9,	5
	*t*≥0	
	cor(*X*1,*X*2)=0.99, cor(*Y*1,*Y*2)=0	
B	λ_*X*1_(*t*)=λ_*X*2_(*t*)=λ_*Y*1_(*t*)=0.9, *t*≥0,	4
	λ_*Y*2_(*t*)=0.5, *t*≥0	
C	$\lambda_{X1}(t)=\left\{\begin{array}{ll} 0.9, & t\leq 0.5\\ 0.6, & t>0.5 \end{array}\right.$, $\lambda_{X2}(t)=\left\{\begin{array}{ll} 0.7, & t\leq 0.5\\ 0.4, & t>0.5 \end{array}\right.$	4
	$\lambda_{Y1}(t)=\left\{\begin{array}{ll} 0.6, & t\leq 0.5\\ 0.9, & t>0.5 \end{array}\right.$, $\lambda_{Y2}(t)=\left\{\begin{array}{ll} 0.4, & t\leq 0.5\\ 0.7, & t>0.5 \end{array}\right.$	
D	$\lambda_{X1}(t)=\left\{\begin{array}{ll} 0.9, & t\leq 0.5\\ 0.7, & t>0.5 \end{array}\right.$, $\lambda_{X2}(t)=\left\{\begin{array}{ll} 0.4, & t\leq 0.5\\ 0.6, & t>0.5 \end{array}\right.$	4
	$\lambda_{Y1}(t)=\left\{\begin{array}{ll} 0.4, & t\leq 0.5\\ 0.7, & t>0.5 \end{array}\right.$, $\lambda_{Y2}(t)=\left\{\begin{array}{ll} 0.9, & t\leq 0.5\\ 0.6, & t>0.5 \end{array}\right.$	
E	$\lambda_{X1}(t)=\left\{\begin{array}{ll} 0.7, & t\leq 0.4\\ 0.9, & t>0.4 \end{array}\right.$, $\lambda_{X2}(t)=\left\{\begin{array}{ll} 0.6, & t\leq 0.4\\ 0.5, & t>0.4 \end{array}\right.$	4
	$\lambda_{Y1}(t)=\left\{\begin{array}{ll} 0.7, & t\leq 0.4\\ 0.5, & t>0.4 \end{array}\right.$, $\lambda_{Y2}(t)=\left\{\begin{array}{ll} 0.6, & t\leq 0.4\\ 0.9, & t>0.4 \end{array}\right.$	

From Tables 3 and 4, the proposed method had comparable performance to the two individual test statistics. In configurations A and C, it performed significantly better than the two individual tests, even though the sample size of 50 was fairly small. In scenarios where only the marginals differed in one component and the correlation structure was similar, it performed poorly compared to the log‐rank test and the Gehan–Wilcoxon test. In the scenarios for early differences in the hazard functions, it obtained almost the performance level of Gehan–Wilcoxon, while the log‐rank test lost a lot of power. For late differences in the hazards, it was the opposite. As an additional comparison between the amount of constraints, we performed additional tests on alternative E, where we added a fifth constraint corresponding to the constraint function in Equation (17) with *g*(*t*)≡1. The resulting Type II error dropped in that case from 73.52 to 60.72 in the censored case and from 27.14 to 21.24 in the uncensored case. In the later case it slightly outperformed the log‐rank test. Adding an additional constraint led to a lower error rate in this scenario. All tests performed better when no censoring occurred, yet one could observe similar patterns as in the censored case for the power comparisons.

**Table 3:** Small sample simulation results of Type I error and Type II error in percent with *n*=*m*=50 for a null (N) and for five different alternative survival distribution configurations (A–E) with censored data for three test statistics

	N	A	B	C	D	E
Combined Constraints	4.83	38.42	66.14	70.94	45.90	73.52
Log‐rank	5.62	93.44	46.60	91.48	56.16	57.68
Gehan	5.29	94.24	59.12	91.28	35.04	82.40

**Table 4:** Small sample simulation results of Type I error and Type II error in percent with *n*=*m*=50 for a null (N) and for five different alternative survival distribution configurations (A–E) with uncensored data for three test statistics

	N	A	B	C	D	E
Combined Constraints	5.00	29.32	40.50	43.16	40.10	27.14
Log‐rank	5.60	93.60	26.28	75.74	64.36	21.74
Gehan	4.99	94.68	41.14	93.72	38.84	56.72

Tables 5 and 6 show that similar results were obtained for moderate sample sizes. All test obtained lower error rates than in the small sample case, but the same properties as in the small sample simulations can be noticed. The strengths of the newly proposed test can be seen here even more clearly in scenarios A and C, which correspond to correlation and crossing hazards, respectively.

**Table 5:** Moderate sample simulation results of Type I error and Type II error in percent with *n*=*m*=150 for a null (N) and for five different alternative survival distribution configurations (A–E) with censored data for three test statistics

	N	A	B	C	D	E
Combined Constraints	4.99	29.76	15.00	22.00	2.82	18.46
Log‐rank	5.38	94.16	3.90	85.54	10.68	8.12
Gehan	5.22	94.10	10.82	83.92	0.86	53.62

**Table 6:** Moderate sample simulation results of Type I error and Type II error in percent with *n*=*m*=150 for a null (N) and for five different alternative survival distribution configurations (A–E) with uncensored data for three test statistics

	N	A	B	C	D	E
Combined Constraints	5.40	25.58	0.98	1.38	1.82	0.06
Log‐rank	5.22	94.00	0.40	34.38	20.60	0.12
Gehan	5.23	93.92	2.28	91.08	1.44	10.00

### 5.1 Data example

In order to illustrate our results on a real dataset, we chose the dataset *retinopathy* from the R‐package *survival*. This package contains the core survival analysis routines. The dataset includes 394 observations out of a total of 197 patients from the Diabetic Retinopathy Study, which was conducted by the National Eye Institute. The observed data was part of a trial on laser coagulation as a treatment to delay diabetic retinopathy. Each patient received laser treatment on one of their eyes, while the other remained untreated to obtain a baseline. The eye for treatment was randomly chosen. The two observations per patient contain some basic information which is the same in both and the time to vision loss in the treated and untreated eye, as well as the risk factor for each eye. The time to vision loss was measured from initiation of treatment to the time when visual acuity dropped below 5/200 for two visits in a row. As no event could occur within the first 6.5 months of the study, all survival times were reduced by this duration. The survival times were subject to univariate censoring. All patients were considered to have "high‐risk" for diabetic retinopathy as defined by the Diabetic Retinopathy Study.

The patients can be split into two groups, *juvenile* and *adult* diabetes. As the types of diabetes are assumed to have differing progress, it is of interest to analyze the joint survival function of both eyes. The null hypothesis which was analyzed in the following was that both juvenile as well as adult diabetes had the same joint survival function of both eyes. Denote the times to blindness in the treated eye by *X*1 for the juvenile group and by *Y*1 for the adult group. Further let *X*2 be the survival time of the untreated eyes in the juvenile group and let *Y*2 be the time in the adult group. With 114 *juvenile* patients and 83 *adult* patients, we have enough observations to narrow the error rate. Censoring rates were 68*%* for *X*1, 55*%* for *X*2, 78*%* for *Y*1, and 40*%* for *Y*2. In the juvenile group 21*%* of the patients could be completely observed, in the adult group 17*%* of the patients.

For the newly proposed method, the constraints (15) and (16) were chosen. In a second calculation we added a fifth constraint, corresponding to Equation (17) with *g*(*t*)=1 applied to the second component.

**Figure 4:**
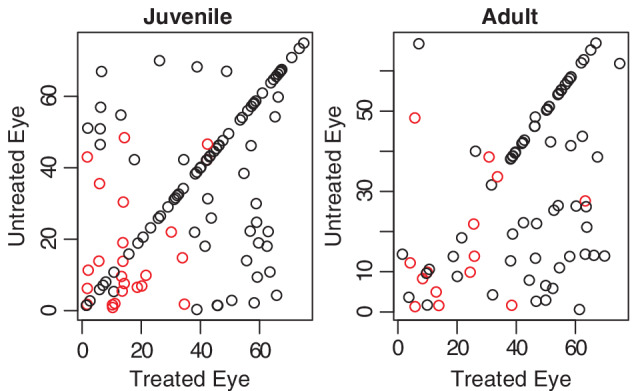
Scatter plot of the two groups. Observations which were completely observed were highlighted in red [Colour figure can be viewed at wileyonlinelibrary.com]

Both tests did not reject the null hypothesis at level 5*%*. The *p* values of the new test method were given by *p*_1_=.2297 and *p*_2_=.1733. Former analyses by Huang and Zhao (2018) and Huster, Brookmeyer, and Self (1989) concluded that the null hypothesis should be rejected. However, both did not actually conduct tests on the similarity of the survival functions. Huster et al. (1989) conducted tests on the similarities of the covariates and rejected the null hypothesis of equal influence of the covariates, while Huang and Zhao (2018) only conducted some visual analysis of the individual estimators of the survival function and their confidence intervals. By examining Figure 4, not rejecting the null hypothesis does not seem entirely unreasonable.

The proposed test tends to be conservative for very small sample sizes and small sample sizes with a high censoring rate. This could already be observed in the univariate case.

## 6 CONCLUSION

On the basis of a previous univariate approach based on empirical likelihood, we developed a multivariate testing procedure for hazards in the survival context with censored observations. Using constraints and conditional hazards, our method is capable of testing various aspects of a dataset containing time‐to‐event observations. It was shown that our proposed method is asymptotically consistent. The finite sample simulation studies further showed that the power of the proposed method depends on the underlying power associated with the individual constraints. For higher sample sizes, it has approximately the power of the better underlying individual test. In scenarios in which the greatest difference of two datasets lies in the correlation structure, the proposed test outperformed the individual tests from the family of test statistics introduced by Harrington and Fleming (1982).

## APPENDIX A PROOF OF THE ONE‐SAMPLE CASE

### A1. Regularity specification for the one‐sample case

Let *X*_1_,…,*X*_*n*_ be i.i.d. *d*‐dimensional random variables with cumulative distribution function *F*_*X*0_(*t*) and marginals *F*_*X*01_(*t*),…,*F*_*X*0*d*_(*t*). The cumulative hazard function is denoted as Λ_*X*0_(*t*) and the marginals as Λ_*X*01_(*t*),…,Λ_*X*0*d*_(*t*). We observe the *d*‐dimensional vectors $T_{i}=\min(X_{i},C_{i}^{X})$ and $\delta_{i}=1\{X_{i}\leq C_{i}^{X}\}$, where $C_{i}^{X}$ are i.i.d. censoring times, independent of *X*_*i*_, and the minimum and the indicator function are considered component‐wise. The cumulative distribution function of the $C_{i}^{X}$ is *F*_*CX*_(*t*). The distribution functions *F*_*X*0_(*t*) and *F*_*CX*_(*t*) do not have common discontinuities.

Let *g*_*j*1_(*t*),…,*g*_*jk*_(*t*), *j*=1,…,*d*, be nonnegative left continuous functions such that none can be expressed by a linear combination of the remaining functions. Further it shall hold for all functions

$$0<\int \frac{\left|g_{jr}(t){|}^{w}(1-\Delta \Lambda_{X0j}(t)\right)}{\left(1-F_{X0j}(t))\underset{j}{\prod }(1-F_{CXj}(t)\right)}d\Lambda_{X0j}(t)<\infty ,\;w=1,2,\;j=1,\ldots ,d,\;r=1,\ldots ,k.$$

This condition guarantees the asymptotic normality of the Nelson–Aalen estimators of the individual components (cf. Gill, 1983, theorem 2.1). Note that the functions *g*_*jr*_(*t*) may be random, yet they need to be predictable w.r.t. a filtration. Considering *d*=2, the filtration is given by $F_{t}=\sigma \{T_{1l}1\{T_{1l}\leq t\};\delta_{1l}1\{T_{1l}\leq t\};T_{2l}1\{T_{2l}\leq t\};\delta_{2l}1\{T_{2l}\leq t\};l=1,\ldots ,n\}$. Through this we are capable of applying the martingale central limit theorem, as ${\hat{\Lambda }}_{NA,j}^{\left(X\right)}(t)-\Lambda_{X0j}(t)$ is a martingale, where ${\hat{\Lambda }}_{NA,j}^{\left(X\right)}$ denotes the Nelson–Aalen estimator of component *j*. Furthermore, for all random functions *g*_*jr*_(*t*) there must exist a nonrandom left continuous function *g*_*jr*0_(*t*) such that $\underset{t\leq \max T_{ji}}{\sup}|g_{jr}(t)/g_{jr0}(t)|=o_{p}(1)$ for *j*=1,…,*d* and *r*=1,…,*k* as *n*→∞.

### A2. Preparation of the proof of Theorem 3

Let *d*=2 and *f*(***λ***) be given by

(A1) $$\begin{array}{ll} & \underset{l}{\sum }\log(1-a_{l}(\lambda_{1}))u_{1l}+\log(a_{l}(\lambda_{1}))u_{2l}+\log(1-b_{l}(\lambda_{2}))u_{3l}+\log(b_{l}(\lambda_{2}))u_{4l}\\ & \;+\log(1-c_{l}(\lambda_{1}))u_{5l}+\log(c_{l}(\lambda_{1}))u_{6l}+\log(1-d_{l}(\lambda_{2}))u_{7l}+\log(d_{l}(\lambda_{2}))u_{8l}, \end{array}$$

with $\lambda ={\left(\lambda_{1}^{T},\lambda_{2}^{T}\right)}^{T}$, ***λ***_*j*_=(λ_*j*1_,…,λ_*jk*_)^*T*^, *j*=1,2, *k* the number of constraints per component, and *l* ranging over all distinct time points an event occurred excluding again the last one as it would be 1. For cases where a hazard is zero we will define the corresponding summand to be zero. Note that we will drop the indices of ***λ*** if it is clear from the context whether ***λ***_1_ or ***λ***_2_ is considered.

In order to show that the NPMLEs of *L* are indeed the maximums we compute the derivatives of *f* w.r.t. all λs and evaluate it at zero.

Without loss of generality we derive *f* only for λ_1*r*_, *r*=1,…*k*, then

$$\begin{array}{ll} \frac{\partial }{\partial \lambda_{1r}}f(\lambda ) & =\underset{l}{\sum }\frac{u_{1l}}{1-a_{l}(\lambda )}\frac{\partial (1-a_{l}(\lambda ))}{\partial \lambda_{1r}}+\frac{u_{2l}}{a_{l}(\lambda )}\frac{\partial a_{l}(\lambda )}{\partial \lambda_{1r}}+\frac{u_{5l}}{1-c_{l}(\lambda )}\frac{\partial (1-c_{l}(\lambda ))}{\partial \lambda_{1r}}+\frac{u_{6l}}{c_{l}(\lambda )}\frac{\partial c_{l}(\lambda )}{\partial \lambda_{1r}}\\ & =\underset{l}{\sum }\left(\frac{-u_{1l}}{1-a_{l}(\lambda )}+\frac{u_{2l}}{a_{l}(\lambda )}\right)\frac{\partial a_{l}(\lambda )}{\partial \lambda_{1r}}+\left(\frac{-u_{5l}}{1-c_{l}(\lambda )}+\frac{u_{6l}}{c_{l}(\lambda )}\right)\frac{\partial c_{l}(\lambda )}{\partial \lambda_{1r}}. \end{array}$$

Evaluating this at ***λ***=**0** we obtain

$$\begin{array}{ll} \frac{\partial }{\partial \lambda_{1r}}f(\lambda ){|}_{\lambda =0} & =\underset{l}{\sum }\left(-\frac{u_{1l}}{\frac{u_{1l}}{u_{1l}+u_{2l}}}+\frac{u_{2l}}{\frac{u_{2l}}{u_{1l}+u_{2l}}}\right)\frac{\partial a_{l}(\lambda )}{\partial \lambda_{1r}}+\left(-\frac{u_{5l}}{\frac{u_{5l}}{u_{5l}+u_{6l}}}+\frac{u_{6l}}{\frac{u_{6l}}{u_{5l}+u_{6l}}}\right)\frac{\partial c_{l}(\lambda )}{\partial \lambda_{1r}}\\ & =\underset{l}{\sum }0\cdot \frac{\partial a_{l}(\lambda )}{\partial \lambda_{1r}}+0\cdot \frac{\partial c_{l}(\lambda )}{\partial \lambda_{1r}}=0. \end{array}$$

The derivation w.r.t. the second component follows analogously. Computing the second derivative *f* ^*′′*^(**0**)=:*D* we can already see from the first derivative that if we take partial derivatives of *f*(***λ***) w.r.t. λ_1*r*_ and then λ_2*s*_, *r*,*s*=1,…,*k* we will obtain zero for all values of ***λ***. Thus we will only calculate it for λ_1*r*_ and λ_1*s*_, *r*,*s*=1,…,*k*. The 1*r*×1*s*‐th element of the 2*k*×2*k* matrix *D* is given by

$$\begin{array}{ll} D_{1r\;\times \;1s}=\frac{\partial^{2}}{\partial \lambda_{1r}\partial \lambda_{1s}}f(\lambda ){|}_{\lambda =0}\;. & \end{array}$$

Denote $u_{1l}+u_{2l}+z_{1l}n\lambda_{1}^{T}G_{1}(t_{1l})$ by *γ*_1*l*_ and $u_{5l}+u_{6l}+z_{3l}n\lambda_{1}^{T}G_{1}(t_{1l})$ by *γ*_3*l*_. Now the second derivative is given by

$$\begin{array}{ll} & \frac{\partial }{\partial \lambda_{1s}}\left[\underset{l}{\sum }\left(\frac{-u_{1l}}{1-a_{l}(\lambda )}+\frac{u_{2l}}{a_{l}(\lambda )}\right)\left(-u_{2l}z_{1l}ng_{1r}\gamma_{1}^{-2}\right)+\left(\frac{-u_{5l}}{1-c_{l}(\lambda )}+\frac{u_{6l}}{c_{l}(\lambda )}\right)(-u_{6l}z_{3l}ng_{1r}\gamma_{3}^{-2})\right]\\ & \;=\underset{l}{\sum }u_{2l}z_{1l}\;ng_{1r}\frac{\partial }{\partial \lambda_{1s}}\left[\left(\frac{u_{1l}}{u_{1l}+z_{1l}n\lambda_{1}^{T}G_{1}(t_{1l})}-\frac{u_{2l}}{u_{2l}}\right)\gamma_{1}^{-1}\right]\\ & \;+\underset{l}{\sum }u_{6l}z_{3l}\;ng_{1r}\frac{\partial }{\partial \lambda_{1s}}\left[\left(\frac{u_{5l}}{u_{5l}+z_{3l}n\lambda_{1}^{T}G_{1}(t_{1l})}-\frac{u_{6l}}{u_{6l}}\right)\gamma_{3}^{-1}\right]\\ & \;=\underset{l}{\sum }u_{2l}z_{1l}^{2}\;n^{2}g_{1r}g_{1s}\gamma_{1}^{-1}(-u_{1l}^{-1})+\underset{l}{\sum }u_{6l}z_{3l}^{2}n^{2}g_{1r}g_{1s}\gamma_{3}^{-1}(-u_{5l}^{-1}). \end{array}$$

For ***λ***=**0** we then obtain

$$D_{1r\;\times \;1s}=-\underset{l}{\sum }\left(\frac{u_{2l}}{u_{1l}}\frac{z_{1l}^{2}n^{2}g_{1r}g_{1s}}{u_{1l}+u_{2l}}+\frac{u_{6l}}{u_{5l}}\frac{z_{3l}^{2}n^{2}g_{1r}g_{1s}}{u_{5l}+u_{6l}}\right).$$

Using the fact that *z*_1*l*_/(*u*_1*l*_+*u*_2*l*_)=*z*_3*l*_/(*u*_5*l*_+*u*_6*l*_) we can rearrange *D*_1*r*×1*s*_ to

$$-\underset{l}{\sum }n^{2}g_{1r}g_{1s}\frac{z_{1l}}{u_{1l}+u_{2l}}\left(\frac{u_{2l}}{u_{1l}}z_{1l}+\frac{u_{6l}}{u_{5l}}z_{3l}\right).$$

The values of *D*_2*r*×2*s*_, *r*,*s*=1,…,*k* can be calculated analogously. Then *D* is the block diagonal matrix

$$D=\left(\begin{array}{cc} D_{1\times 1} & 0\\ 0 & D_{2\times 2} \end{array}\right),$$

where *D*_1×1_ is the matrix with the entries *D*_1*r*×1*s*_, *r*,*s*=1,…,*k* and *D*_2×2_ analogously. By a now standard counting process martingale assumption we see that the elements of −*D*/*n* converge in probability to the elements of *D* ^∗^.

Define

$$Y_{1l}:=\frac{\delta_{1l}nz_{1}(T_{1l}){\tilde{\lambda }}_{1}^{T}G_{1}(T_{1l})}{u_{1l}(T_{1l})+u_{2l}(T_{1l})}\;\text{and}\;Y_{3l}:=\frac{\delta_{1l}nz_{3}(T_{1l}){\tilde{\lambda }}_{1}^{T}G_{1}(T_{1l})}{u_{5l}(T_{1l})+u_{6l}(T_{1l})},\;l=1,\ldots ,n,$$

where *z*_1_(*T*_1*l*_) is simply $z_{1l^{*}}$ with $t_{1l^{*}}=T_{1l}$, analogously for *z*_3_(*T*_1*l*_). As *T*_1*l*_ are defined to be i.i.d. we can conclude that *Y*_1*l*_ and *Y*_3*l*_ are i.i.d. as well. Due to the regularity specification it holds that $E(Y_{1i}^{\;2})<\infty$ and $E(Y_{3i}^{\;2})<\infty$.

Lemma 3
*Let Y*_1*i*_ *and Y*_3*i*_ *be as above. Under the regularity specifications it holds that*

(A2) $$1/n\overset{n}{\underset{i=1}{\sum }}(Y_{1i}^{\;2}+Y_{3i}^{\;2})$$

*converges to*

$$\int \frac{{\left({\tilde{\lambda }}_{1}^{T}G_{1}\right)}^{2}d\Lambda_{X01}}{\left(1-F_{X01})\underset{j}{\prod }(1-F_{CXj}\right)}.$$

*The convergence is of order O*_*p*_(1).

The sum in Equation (A2) can be rearranged to

$$\underset{t_{1l}}{\sum }{\left({\tilde{\lambda }}_{1}^{T}G_{1}(t_{1l})\right)}^{2}(u_{2l}+u_{6l})\frac{n}{u_{1l}+u_{2l}+u_{5l}+u_{6l}}\delta_{1l}.$$

Define $Z(t)=\sum_{i=1}^{n}1\{T_{1i}\geq t\}$. Then the term *u*_1*l*_+*u*_2*l*_+*u*_5*l*_+*u*_6*l*_ can be replaced by $\hat{Z}(t_{1l})$. Further δ_1*l*_=1 if *u*_2*l*_+*u*_6*l*_≠0 and zero otherwise. We obtain

$$\underset{t_{1l}}{\sum }\frac{{\left({\tilde{\lambda }}_{1}^{T}G_{1}(t_{1l})\right)}^{2}}{\hat{Z}(t_{1l})/n}(u_{2l}+u_{6l}).$$

Now we can rewrite this in terms of the Nelson–Aalen estimator,

$$\int_{0}^{\infty }\frac{{\left({\tilde{\lambda }}_{1}^{T}G_{1}(t)\right)}^{2}}{\hat{Z}(t)/n}d{\hat{\Lambda }}_{NA,1},$$

where ${\hat{\Lambda }}_{NA,1}$ denotes the Nelson‐Aalen estimator of the first component. Analogue to Lemma 4.1 of Dabrowska (1988) the supremum norm of the component‐wise cumulative hazard function converges to zero, which follows from the Glivenko–Cantelli Theorem and simple algebra under the regularity specifications. Applying Lenglart's inequality and using lemma 3.1.3.9 of Spreij (1990) we can switch the integral w.r.t. the true component‐wise cumulative hazard function. Further, the empirical distribution given through $\hat{Z}(t)/n$ converges uniformly to (1−*F*_*X*01_)(1−*F*_*CX*_). Thus the convergence of the sum holds true.

Lemma 4
*Define* $Z_{n}:=\underset{1\leq i\leq n}{\max}Y_{1i}$ *, with Y*_1*i*_ *i.i.d.random variables, and* $E(Y_{1i}^{2})<\infty$ *. It follows that Z*_*n*_=*o*_*p*_(*n* ^1/2^) *a.s. as n*→∞.

This follows from lemma A1 of Pan and Zhou (2002).

Lemma 5
*Assume the data are such that the NPMLEs of EL are asymptotically normal and the variance–covariance matrix* ∑ *defined in the proof is invertible. Then for the solution* $\lambda_{X}={\left(\lambda_{1}^{T},\lambda_{2}^{T}\right)}^{T}$ *of the constrained problem (* 9 *), corresponding to the null hypothesis H*_0_ *, as given in Equation (* 4 *), it holds that n* ^1/2^ ***λ***_*X*_ *converges in distribution to N*(0,∑).

Define ***ϕ***(*s*)=(*ϕ*_11_(*s*_1_),…,*ϕ*_1*k*_(*s*_1_),*ϕ*_21_(*s*_2_),…,*ϕ*_2*k*_(*s*_2_)) with $\phi_{jr}(t_{ji})=\sum_{i}g_{jr}(t_{ji})\log(1-v_{ji}(s_{j}))-\mu_{jr}$ for *j*=1,2, *r*=1,…,*k* and $s={\left(s_{1}^{T},s_{2}^{T}\right)}^{T}\in R^{2k}$. Then ***λ***_*X*_ is the unique solution of ***ϕ***(***s***)=0 under the regularity specification. Thus we can expand the term ***ϕ***(***λ***) to ***ϕ***(**0**)+***ϕ***′(**0**)***λ***+*o*_*p*_(*n* ^−1/2^), where ***ϕ***′(**0**) is a 2*k*×2*k* matrix. The next part proves that this indeed holds true. Rewrite $\lambda_{j}=p_{j}{\tilde{\lambda }}_{j}$, where $\|{\tilde{\lambda }}_{j}\|=1$, *j*=1,2. Then,

$$\begin{array}{ll} 0={\tilde{\lambda }}^{T}\phi (\lambda ) & =\underset{l}{\sum }{\tilde{\lambda }}_{1}^{T}G_{1}(t_{1l})\log(1-v_{1l}(\lambda_{1}))-{\tilde{\lambda }}_{1}^{T}\mu_{1}\\ & \;+\underset{l}{\sum }{\tilde{\lambda }}_{2}^{T}G_{2}(t_{2l})\log(1-v_{2l}(\lambda_{2}))-{\tilde{\lambda }}_{2}^{T}\mu_{2}. \end{array}$$

We can split these terms up in a part without the Lagrange multiplier and a part with,

$$\begin{array}{ll} & \underset{l}{\sum }{\tilde{\lambda }}_{1}^{T}G_{1}(t_{1l})\log(1-v_{1l}(0))-{\tilde{\lambda }}_{1}^{T}\mu_{1}\;(i)\\ & \;+\underset{l}{\sum }\left({\tilde{\lambda }}_{1}^{T}G_{1}(t_{1l})[log(1-v_{1l}(\lambda_{1}))-\log(1-v_{1l}(0))]\right)\;(ii)\\ & \;+\underset{l}{\sum }{\tilde{\lambda }}_{2}^{T}G_{2}(t_{2l})\log(1-v_{2l}(0))-{\tilde{\lambda }}_{2}^{T}\mu_{2}\;(iii)\\ & \;+\underset{l}{\sum }\left({\tilde{\lambda }}_{2}^{T}G_{2}(t_{2l})[log(1-v_{2l}(\lambda_{2}))-\log(1-v_{2l}(0))]\right)\;(iv). \end{array}$$

Again we will only consider the terms (i) and (ii) in the following as the other terms follow analogously. It holds that term (*i*) is of order *o*_*p*_(*n* ^−1/2^) under the null hypothesis. This follows directly from the properties of the NPMLEs. The term (*ii*) can be split into the two terms

$$B_{1}=\underset{l}{\sum }{\tilde{\lambda }}_{1}^{T}G_{1}(t_{1l})z_{1l}\left[\log(1-a_{l}(\lambda_{1}))-\log(1-a_{l}(0))\right],$$

and

$$B_{2}=\underset{l}{\sum }{\tilde{\lambda }}_{1}^{T}G_{1}(t_{1l})z_{3l}\left[\log(1-c_{l}(\lambda_{1}))-\log(1-c_{l}(0))\right],$$

with an error of order $o_{p}(\max{\;v}_{1l})$. We will rearrange and estimate *B*_1_. It holds, when applying $\left|\varepsilon_{1}-\varepsilon_{2}|\leq |\;\log\;(\varepsilon_{1})-\log(\varepsilon_{2})\right|$ for *ε*_1_,*ε*_2_∈(0,1], that

$$\begin{array}{ll} \left|B_{1}\right| & \geq \left|\underset{l}{\sum }{\tilde{\lambda }}_{1}^{T}G_{1}(t_{1l})z_{1l}\left[1-a_{l}(\lambda_{1})-1+a_{l}(0)\right]\right|\\ & =\left|\underset{l}{\sum }z_{1l}{\tilde{\lambda }}_{1}^{T}G_{1}(t_{1l})\frac{u_{2l}z_{1l}n\lambda_{1}^{T}G_{1}(t_{1l})}{\left(u_{1l}+u_{2l})(u_{1l}+u_{2l}+z_{1l}n\lambda_{1}^{T}G_{1}(t_{1l})\right)}\right|\\ & =\left|\underset{l}{\sum }\frac{p_{1}nu_{2l}{\left(z_{1l}{\tilde{\lambda }}_{1}^{T}G_{1}(t_{1l})\right)}^{2}}{{\left(u_{1l}+u_{2l}\right)}^{2}+{\left(u_{1l}+u_{2l}\right)}^{2}\;\frac{z_{1l}p_{1}n{\tilde{\lambda }}_{1}^{T}G_{1}(t_{1l})}{u_{1l}+u_{2l}}}\right|\;. \end{array}$$

Analogously for *B*_1_ and *B*_2_ we obtain in total

$$\begin{array}{ll} \left|B_{1}+B_{2}\right| & \geq |\underset{l}{\sum }\frac{p_{1}nu_{2l}{\left(z_{1l}{\tilde{\lambda }}_{1}^{T}G_{1}(t_{1l})\right)}^{2}}{{\left(u_{1l}+u_{2l}\right)}^{2}+{\left(u_{1l}+u_{2l}\right)}^{2}\;\frac{z_{1l}p_{1}n{\tilde{\lambda }}_{1}^{T}G_{1}(t_{1l})}{u_{1l}+u_{2l}}}\\ & \;+\underset{l}{\sum }\frac{p_{1}nu_{6l}{\left(z_{3l}{\tilde{\lambda }}_{1}^{T}G_{1}(t_{1l})\right)}^{2}}{{\left(u_{5l}+u_{6l}\right)}^{2}+{\left(u_{5l}+u_{6l}\right)}^{2}\;\frac{z_{3l}p_{1}n{\tilde{\lambda }}_{1}^{T}G_{1}(t_{1l})}{u_{5l}+u_{6l}}}|\\ & =|\underset{l}{\sum }[\frac{z_{1l}^{2}}{{\left(u_{1l}+u_{2l}\right)}^{2}}\frac{p_{1}nu_{2l}{\left({\tilde{\lambda }}_{1}^{T}G_{1}(t_{1l})\right)}^{2}}{1+\frac{z_{1l}}{u_{1l}+u_{2l}}p_{1}n{\tilde{\lambda }}_{1}^{T}G_{1}(t_{1l})}\\ & \;+\frac{z_{3l}^{2}}{{\left(u_{5l}+u_{6l}\right)}^{2}}\frac{p_{1}nu_{6l}{\left({\tilde{\lambda }}_{1}^{T}G_{1}(t_{1l})\right)}^{2}}{1+\frac{z_{3l}}{u_{5l}+u_{6l}}p_{1}n{\tilde{\lambda }}_{1}^{T}G_{1}(t_{1l})}]|. \end{array}$$

As it holds that *z*_1*l*_/(*u*_1*l*_+*u*_2*l*_)=*z*_3*l*_/(*u*_5*l*_+*u*_6*l*_), we can merge the two sums and obtain

$$\begin{array}{ll} & \left|\underset{l}{\sum }\frac{p_{1}}{1+p_{1}n\frac{z_{1l}}{u_{1l}+u_{2l}}{\tilde{\lambda }}_{1}^{T}G_{1}(t_{1l})}\;\frac{z_{1l}^{2}n(u_{2l}+u_{6l}){\left({\tilde{\lambda }}_{1}^{T}G_{1}(t_{1l})\right)}^{2}}{{\left(u_{1l}+u_{2l}\right)}^{2}}\right|\\ & \;\geq \frac{\left|p_{1}\right|}{1+|p_{1}|n\underset{l}{\;\max}\;\frac{z_{1l}{\tilde{\lambda }}_{1}^{T}G_{1}(t_{1l})}{u_{1l}+u_{2l}}}\underset{l}{\sum }\frac{{\left(z_{1l}{\tilde{\lambda }}_{1}^{T}G_{1}(t_{1l})\right)}^{2}n(u_{2l}+u_{6l})}{{\left(u_{1l}+u_{2l}\right)}^{2}}. \end{array}$$

From Lemma 3 it follows that the sum in the last expression is of order *O*_*p*_(1), under the regularity specification the requirements for Lemma 4 are fulfilled and the maximum in the denominator is of order *o*_*p*_(*n* ^1/2^). Hence |*p*_1_| is of order *O*_*p*_(*n* ^−1/2^). The proof for the remaining terms follows analogously and the expansion above is valid.

Therefore,

$$n^{1/2}\lambda =\phi^{{\;}^{\prime }}{\left(0\right)}^{-1}(-n^{1/2}\phi (0))+o_{p}(1).$$

The elements $\phi_{rs}^{\prime }$ of ***ϕ***′(**0**) can easily be computed, and are equivalent to −*D*_*rl*_/*n* where *r*,*l*=1,…,2*k*. By the counting process martingale central limit theorem, we can show that *n* ^1/2^ ***λ*** converges in distribution to *N*(**0**,∑_*ϕ*_) with $\sum_{\phi }=\lim{\;\phi }^{\prime }(0)$. All together we obtain that *n* ^1/2^ ***λ*** converges in distribution to *N*(**0**,∑) with $\sum :=\lim\;{\left(\phi^{\prime }(0)\right)}^{-1}$. Note that ∑^−1^=*D* ^∗^.

### A3. Proof of Theorem 3

Let *f*(***λ***) be defined as in Equation (A1), then *W*=−2(*f*(***λ***_*x*_)−*f*(**0**)), where ***λ***_*x*_ denotes the Lagrange multiplier of the optimum. By Taylor expansion, which is valid as ***λ***_*x*_ is close to zero, we obtain

$$W=2\{f(0)-f(0)-f^{\prime }(0)\lambda_{x}-\frac{1}{2}\lambda_{x}^{T}D\lambda_{x}+o_{p}(1)\},$$

where *D* is again the matrix of the second derivative of *f*(***λ***) w.r.t. ***λ***. From the preparation part we have *f*′(**0**)=0 and we can simplify *W* to $-\lambda_{x}^{T}\sum \lambda_{x}+o_{p}(1)$. Note that −∑ is symmetric and positive definite for large enough *n*. This follows from the convergence of −*D*/*n* to a positive definite matrix *D* ^∗^=∑^−1^, see proof of Lemma 5. Now $W=\lambda_{x}^{T}{\left(-D\right)}^{1/2}{\left(-D\right)}^{1/2}\lambda_{x}+o_{p}(1)$ and as $n^{1/2}\lambda_{x}^{T}(D^{1/2}n^{-1/2})$ converges in distribution to *N*(**0**,*I*), we obtain that W converges in distribution to $X_{2k}^{2}$.

## APPENDIX B PROOF OF THE TWO‐SAMPLE CASE

### B1. Regularity specification for the two‐sample case

Let *X*_1_,…,*X*_*n*_ be i.i.d. *d*‐dimensional random variables as already defined in Section A.1. Let *Y*_1_,…,*Y*_*n*_ be i.i.d. *d*‐dimensional random variables with cumulative distribution function *F*_*Y*0_(*t*) and marginals *F*_*Y*01_(*t*),…,*F*_*Y*0*d*_(*t*). The cumulative hazard function is denoted by Λ_*Y*0_(*t*) and the marginals by Λ_*Y*01_(*t*),…,Λ_*Y*0*d*_(*t*). We observe the *d*‐dimensional vectors $T_{i}=\min(X_{i},C_{i}^{X})$, $\delta_{i}=1\{X_{i}\leq C_{i}^{X}\}$, $S_{i}=\min(Y_{i},C_{i}^{Y})$ and $\tau_{i}=1\{Y_{i}\leq C_{i}^{Y}\}$ where $C_{i}^{X}$ and $C_{i}^{Y}$ are i.i.d. censoring times, independent of *X*_*i*_ and *Y*_*i*_, and the minimum and the indicator function are considered component‐wise. The cumulative distribution function of the $C_{i}^{X}$ is given by *F*_*CX*_(*t*) and for $C_{i}^{Y}$ it is *F*_*CY*_(*t*). The distribution functions *F*_*X*0_(*t*) and *F*_*CX*_(*t*) do not have common discontinuities, respectively, the same holds for the distribution functions *F*_*Y*0_(*t*) and *F*_*CY*_(*t*).

Let *g*_*j*1_(*t*),…,*g*_*jk*_(*t*),*h*_*j*1_(*t*),…,*h*_*jk*_(*t*), *j*=1,…,*d*, be nonnegative left continuous functions such that none can be expressed by a linear combination of the rest of the functions. Further it shall hold for all functions

$$0<\int \frac{\left|g_{jr}(t){|}^{w}(1-\Delta \Lambda_{X0j}(t)\right)}{\left(1-F_{X0j}(t))\underset{j}{\prod }(1-F_{CXj}(t)\right)}d\Lambda_{X0j}(t)<\infty ,$$

and

$$0<\int \frac{\left|h_{jr}(t){|}^{w}(1-\Delta \Lambda_{Y0j}(t)\right)}{\left(1-F_{Y0j}(t))\underset{j}{\prod }(1-F_{CYj}(t)\right)}d\Lambda_{Y0j}(t)<\infty ,$$

with *w*=1,2,*j*=1,…,*d*,*r*=1,…,*k*. Note that the functions *g*_*jr*_(*t*) and *h*_*jr*_(*t*) may be random, yet they need to be predictable w.r.t. a filtration. For *d*=2 the filtration is given by

(B1) $$\begin{array}{ll} F_{t} & =\sigma \{T_{1l}1\{T_{1l}\leq t\};\delta_{1l}1\{T_{1l}\leq t\};T_{2l}1\{T_{2l}\leq t\};\delta_{2l}1\{T_{2l}\leq t\};\\ & \;\;\;S_{1l}1\{S_{1l}\leq t\};\tau_{1l}1\{S_{1l}\leq t\};S_{2l}1\{S_{2l}\leq t\};\tau_{2l}1\{S_{2l}\leq t\};l=1,\ldots ,n\}. \end{array}$$

Through this we are capable of applying the martingale central limit theorem, as ${\hat{\Lambda }}_{NA,j}^{\left(X\right)}(t)-\Lambda_{X0j}(t)$ is then a martingale, where ${\hat{\Lambda }}_{NA,j}^{\left(X\right)}$ denotes the Nelson–Aalen estimator of component *j* for samples of *X*, respectively, for ${\hat{\Lambda }}_{NA,j}^{\left(Y\right)}(t)-\Lambda_{Y0j}(t)$.

Furthermore, for all random functions *g*_*jr*_(*t*) and *h*_*jr*_(*t*) there must exist a nonrandom left continuous function *g*_*jr*0_(*t*) and *h*_*jr*0_(*t*), respectively, such that

$$\underset{t\leq \max T_{ji}}{\sup}|g_{jr}(t)/g_{jr0}(t)|=o_{p}(1)\;\text{and}\;\underset{t\leq \max S_{ji}}{\sup}|h_{jr}(t)/h_{jr0}(t)|=o_{p}(1)$$

for *j*=1,…,*d* and *r*=1,…,*k* as *n*→∞.

### B2. Mathematical derivations and proof of Theorem 5

The constraints are defined as in Equation (12). Define ***G***_*j*_(*t*_*i*_)=(*g*_*j*1_(*t*_*i*_),…,*g*_*jk*_(*t*_*i*_))^*T*^ and ***H***_*j*_(*s*_*i*_)=(*h*_*j*1_(*s*_*i*_),…,*h*_*jk*_(*s*_*i*_))^*T*^, *j*=1,2, the vectors of constraint‐function values. By application of the Lagrange multiplier method we know that the maximum‐likelihood estimators are given by the NPMLE as defined in Equation (13).

The function *f*(***λ***), similar to Equation (A1), is given by

$$\begin{array}{ll} & \underset{l}{\sum }\log(1-a_{l}(\lambda_{1}))u_{1l}+\log(a_{l}(\lambda_{1}))u_{2l}+\log(1-b_{l}(\lambda_{2}))u_{3l}+\log(b_{l}(\lambda_{2}))u_{4l}\\ & \;+\log(1-c_{l}(\lambda_{1}))u_{5l}+\log(c_{l}(\lambda_{1}))u_{6l}+\log(1-d_{l}(\lambda_{2}))u_{7l}+\log(d_{l}(\lambda_{2}))u_{8l}\\ & \;+\underset{l}{\sum }\log(1-\alpha_{l}(\lambda_{1}))\nu_{1l}+\log(\alpha_{l}(\lambda_{1}))\nu_{2l}+\log(1-\beta_{l}(\lambda_{2}))\nu_{3l}+\log(\beta_{l}(\lambda_{2}))\nu_{4l}\\ & \;+\log(1-\gamma_{l}(\lambda_{1}))\nu_{5l}+\log(\gamma_{l}(\lambda_{1}))\nu_{6l}+\log(1-\varepsilon_{l}(\lambda_{2}))\nu_{7l}+\log(\varepsilon_{l}(\lambda_{2}))\nu_{8l}. \end{array}$$

Taking the derivatives of this function we obtain that *f*′(**0**)=**0** and *f′′*(**0**)=*D*. The elements of *D* are zero where one first takes the derivative w.r.t λ_1*r*_ and then w.r.t. λ_2*s*_ or vice versa, *r*,*s*=1,…,*k*, that is, *D* is again a block diagonal matrix. The element *D*_1*r*×1*s*_, obtained by taking the derivatives of *f*(***λ***) w.r.t. λ_1*r*_ and λ_1*s*_, *r*,*s*=1,…,*k*, is given by

$$-\left[\underset{l}{\sum }\left(\frac{u_{2l}}{u_{1l}}\frac{z_{1l}^{2}n^{2}g_{1r}g_{1s}}{u_{1l}+u_{2l}}+\frac{u_{6l}}{u_{5l}}\frac{z_{3l}^{2}n^{2}g_{1r}g_{1s}}{u_{5l}+u_{6l}}\right)+\underset{l}{\sum }\left(\frac{\nu_{2l}}{\nu_{1l}}\frac{\zeta_{1l}^{2}n^{2}h_{1r}h_{1s}}{\nu_{1l}+\nu_{2l}}+\frac{\nu_{6l}}{\nu_{5l}}\frac{\zeta_{3l}^{2}n^{2}h_{1r}h_{1s}}{\nu_{5l}+\nu_{6l}}\right)\right].$$

As we assumed that *n*/*m*→*c*∈(0,∞) as min(m,n)→∞ we obtain that the elements of −*D*/*n* converge in probability to the elements of *D* ^∗∗^. Again we need to show the asymptotic normality of *n* ^1/2^ ***λ***_*xy*_, with ***λ***_*xy*_ the solution of the constrained problem (12). Define ***ϕ***(***s***)=(*ϕ*_11_(*s*_1_),…,*ϕ*_1*k*_(*s*_1_),*ϕ*_21_(*s*_2_),…,*ϕ*_2*k*_(*s*_2_)) with

$$\phi_{jr}(s_{j})=\underset{l}{\sum }g_{jr}(t_{jl})\log(1-v_{jl}(s_{j}))-\underset{l}{\sum }h_{jr}(s_{jl})\log(1-w_{jl}(s_{j}))-\mu_{jr}$$

for *j*=1,2, *r*=1,…,*k* and $s={\left(s_{1},s_{2}\right)}^{T}\in R^{2}$ where the two sums are taken over all time points *t*_*jl*_, respectively, *s*_*jl*_, excluding the last.

Set $\lambda_{j}=p_{j}{\tilde{\lambda }}_{j}$ with $||{\tilde{\lambda }}_{j}||=1$, *j*=1,2. Now

$$\begin{array}{ll} 0 & ={\tilde{\lambda }}^{T}\phi (\lambda )=\underset{l}{\sum }{\tilde{\lambda }}_{1}^{T}G_{1}(t_{1l})\log(1-v_{1l}(\lambda_{1}))-\underset{l}{\sum }{\tilde{\lambda }}_{1}^{T}H_{1}(s_{1l})\log(1-w_{1l}(\lambda_{1}))-{\tilde{\lambda }}_{1}^{T}\mu_{1}\\ & \;+\underset{l}{\sum }{\tilde{\lambda }}_{2}^{T}G_{2}(t_{2l})\log(1-v_{2l}(\lambda_{2}))-\underset{l}{\sum }{\tilde{\lambda }}_{2}^{T}H_{2}(s_{2l})\log(1-w_{2l}(\lambda_{1}))-{\tilde{\lambda }}_{2}^{T}\mu_{2}, \end{array}$$

with ***μ***_1_=(*μ*_11_,…,*μ*_1*k*_)^*T*^ and ***μ***_2_=(*μ*_21_,…,*μ*_2*k*_)^*T*^. Splitting this into the four terms *A*_1_, *A*_2_, *B*_1_, and *B*_2_, which are given by

$$\begin{array}{ll} A_{1} & =\underset{l}{\sum }{\tilde{\lambda }}_{1}^{T}G_{1}(t_{1l})\log(1-v_{1l}(0))-\underset{l}{\sum }{\tilde{\lambda }}_{1}^{T}H_{1}(s_{1l})\log(1-w_{1l}(0))-{\tilde{\lambda }}_{1}^{T}\mu_{1}=O_{p}(n^{-1/2}),\\ A_{2} & =\underset{l}{\sum }{\tilde{\lambda }}_{2}^{T}G_{2}(t_{2l})\log(1-v_{2l}(0))-\underset{l}{\sum }{\tilde{\lambda }}_{2}^{T}H_{2}(s_{2l})\log(1-w_{2l}(0))-{\tilde{\lambda }}_{2}^{T}\mu_{2}=O_{p}(n^{-1/2}), \end{array}$$

where the last equation in *A*_1_ and *A*_2_ holds under the null hypothesis,

$$\begin{array}{ll} B_{1} & =\underset{l}{\sum }{\tilde{\lambda }}_{1}^{T}G_{1}(t_{1l})\left[\log(1-v_{1l}(\lambda_{1}))-\log(1-v_{1l}(0))\right]\\ & \;-\underset{l}{\sum }{\tilde{\lambda }}_{1}^{T}H_{1}(s_{1l})\left[\log(1-w_{1l}(\lambda_{1}))-\log(1-w_{1l}(0))\right] \end{array}$$

and

$$\begin{array}{ll} B_{2} & =\underset{l}{\sum }{\tilde{\lambda }}_{2}^{T}G_{2}(t_{2l})[\log(1-v_{2l}(\lambda_{2}))-\log(1-v_{2l}(0))]\\ & \;-\underset{l}{\sum }{\tilde{\lambda }}_{2}^{T}H_{2}(s_{2l})[\log(1-w_{2l}(\lambda_{1}))-\log(1-w_{2l}(0))]. \end{array}$$

For *B*_1_ we obtain, with similar calculations as in the one‐sample case,

$$\begin{array}{ll} \left|B_{1}\right| & \geq \frac{\left|p_{1}\right|}{1+|p_{1}|\min(m,n)\max\left\{\underset{l}{\max}\frac{z_{1l}{\tilde{\lambda }}_{1}^{T}G_{1}(t_{1l})}{u_{1l}+u_{2l}};\underset{l}{\max}\frac{\zeta_{1l}{\tilde{\lambda }}_{1}^{T}H_{1}(s_{1l})}{\nu_{1l}+\nu_{2l}}\right\}}\\ & \;\times \left(\underset{l}{\sum }\frac{{\left(z_{1l}{\tilde{\lambda }}_{1}^{T}G_{1}(t_{1l})\right)}^{2}\min(m,n)(u_{2l}+u_{6l})}{{\left(u_{1l}+u_{2l}\right)}^{2}}+\underset{l}{\sum }\frac{{\left(\zeta_{1l}{\tilde{\lambda }}_{1}^{T}H_{1}(s_{1l})\right)}^{2}\min(m,n)(\nu_{2l}+\nu_{6l})}{{\left(\nu_{1l}+\nu_{2l}\right)}^{2}}\right). \end{array}$$

Again the sum in the last expression is of order *O*_*p*_(1), and |*p*_1_| is of order *O*_*p*_(*n* ^−1/2^). Thus

$$0=\phi (\lambda )=\phi (0)+\phi^{\prime }(0)\lambda +o_{p}(n^{-1/2})$$

and by the counting process martingale central limit theorem we obtain that *n* ^1/2^ ***λ*** converges in distribution to *N*(**0**,∑) with $\sum =\lim{\left\{\phi^{\prime }(0)\right\}}^{-1}$. Note that ***ϕ***′(**0**)=−*D*/*n*.

By Taylor expansion of *W* ^∗^=−2(*f*(***λ***_*xy*_)−*f*(**0**)), as ***λ***_*xy*_ is close to zero, it holds

$$W^{*}=-2(f^{\prime }(0)\lambda_{xy}+\frac{1}{2}\lambda_{xy}^{T}D\lambda_{xy})+o_{p}(1)=\lambda_{xy}^{T}{\left(-D\right)}^{1/2}{\left(-D\right)}^{1/2}\lambda_{xy}+o_{p}(1).$$

Now as $\lambda_{xy}^{T}{\left(-D\right)}^{1/2}$ converges in distribution to *N*(0,1) we obtain the result of Theorem 5.

#### B3. Notes on Proof of Theorem 4 and Theorem 6

Due to notation we will not explicitly give the proof of those two theorems. For a fixed dimension the proof follows analogue to the two‐dimensional case. First defines the function *f* and calculates the derivatives. Second defines the function *ϕ* and calculates, by skillfully splitting it up in several sums, the rates of convergence. Last, one applies a Taylor expansion to obtain the results.
